# Reintroduction modifies the intraspecific variations of symbiotic microbes in captive bred Chinese giant salamander

**DOI:** 10.3389/fmicb.2022.1062604

**Published:** 2022-12-01

**Authors:** Jianyi Feng, Wei Zhu, Jianping Jiang, Chunlin Zhao, Zijian Sun, Wansheng Jiang, Qinghua Luo, Tian Zhao

**Affiliations:** ^1^CAS Key Laboratory of Mountain Ecological Restoration and Bioresource Utilization & Ecological Restoration Biodiversity Conservation Key Laboratory of Sichuan Province, Chengdu Institute of Biology, Chinese Academy of Sciences, Chengdu, China; ^2^University of Chinese Academy of Sciences, Beijing, China; ^3^Hunan Engineering Laboratory for Chinese Giant Salamander's Resource Protection and Comprehensive Utilization, and Key Laboratory of Hunan Forest and Chemical Industry Engineering, Jishou University, Zhangjiajie, China

**Keywords:** alpha and beta diversity, reintroduction, microbiome reorganization, amphibian conservation, *Andrias davidianus*

## Abstract

Microorganisms play as fundamental contributors to maintain hosts’ fitness, which can be shaped by external environment. Moreover, symbiotic microbiome also varied within species (e.g., between sexes and developmental stages). However, we still need more studies to quantify whether the intraspecific variation patterns of symbiotic microbes can be modified with the change of environment. The Chinese giant salamander (CGS; *Andrias davidianus*) is a Critically Endangered species. Despite quantitative captive bred individuals were released to rebuild wild populations, the effectiveness is limited. More importantly, no studies have revealed the adaptation of released CGSs to the complex field conditions. In the present study, we explored whether reintroduction can reshape the intraspecific variations of symbiotic microbiota in captive bred CGSs using high-throughput amplicon sequencing of the16S rRNA gene. We found no significant difference of symbiotic microbiome in captive bred males and females, but released males and females differed significantly in skin microbiome. Juveniles had higher diversity of microbial symbiont than adults in hatchery, but lower diversity in field. Moreover, dominant bacterial taxa differed between juveniles and adults in both hatchery and field. Importantly, this symbiotic microbiome variations within species can be modified (alpha and beta diversity, and community composition) when captive bred individuals were released to the field. Overall, we observed a lower alpha diversity and higher relative abundance of *Chryseobacterium*, *Plesiomonas*, and *Acinetobacter* in the bacterial community of captive bred individuals. Instead, higher alpha diversity of symbiotic microbiota and higher relative abundance of S24-7 and *Lactobacillus* was detected in released individuals. These modifications may associate with the change of living environment, as well as the specific behavior within CGSs (e.g., movement patterns and foraging activities). Future studies can incorporate other approaches (e.g., blood physiology) to better evaluate the growth and health of reintroduced CGSs.

## Introduction

Microorganisms widely exist throughout the whole body sites of animals. They co-evolve with hosts over time, and play as fundamental contributors to maintain hosts’ fitness (Suzuki, 2017; Gould et al., 2018). Specifically, oral cavity acts as a channel connecting the living environment and the digestive tract of host. Accordingly, oral microbial symbionts can protect host against the disturbance of external pathogenic bacteria when feeding (Takahashi, 2015; Gao et al., 2018). Moreover, skin microbiome is considered to be an integral component of hosts immune system, acting as a barrier to improve the disease resistance (Naik et al., 2012; Rebollar et al., 2016). Gut microbes display key functional roles, regulating hosts important physiological processes such as digestion, energy metabolism, pathogen defense, and immunomodulation (Chung et al., 2012; Hu et al., 2018; Raymann and Moran, 2018; Levin et al., 2021; Xia et al., 2022). Therefore, investigating the dynamics of symbiotic bacterial diversity and composition can help us better understand hosts immunity and health.

The composition of host-associated microbial symbiont can be shaped by external environmental conditions (Bletz et al., 2016; Muletz Wolz et al., 2018; Moeller et al., 2020). Therefore, one can expect that the host-associated microbiota should be distinct when organisms occupy different habitats. Accordingly, the symbiotic microbes of captive bred animals should be changed when they were released to the field. For instance, the captive and wild Beal’s eyed turtle (*Sacalia bealei*) displayed different alpha diversity, beta diversity, and community composition of gut microbiota due to different diets and habitat conditions (Fong et al., 2020). Moreover, hosts symbiotic bacteria can be also different between developmental stages and sexes within species (Kueneman et al., 2014; Bennett et al., 2016; Hernandez-Gomez et al., 2018). For instance, the alpha and beta diversity of brown frogs (*Rana dybowskii*) gut bacterial varied significantly between tadpoles and adults, which can be attributed to the shift of diet from plants to arthropod species during metamorphosis (Tong et al., 2020). Similar situation can be detected between male and female Chinese concave-eared frogs (*Odorrana tormota*), which exhibited distinct community composition of gut microbiota. This may be because females have a wider trophic niche size than males (Shu et al., 2019). However, few studies have been conducted to test whether the transition of captive bred individuals from hatchery to the wild will induce the change of intraspecific variations of their symbiotic microbes. Captive bred individuals live in the similar external environment, thus there should be no significant difference of symbiotic microbes between juveniles, males, and females in the hatchery. In contrast, since the field condition is more complex, and released juveniles, males, and females can move freely to select the preferred habitat, their symbiotic microbes should be significantly different.

Chinese giant salamander (CGS; *Andrias davidianus*) is the largest extant amphibian species in the world (Murphy et al., 2000), attracting quantitative attentions of ecologists and biologists for decades (Hou et al., 2004). However, the natural populations of CGS have dramatically declined since 1950s due to habitat loss, environmental contamination, and overexploitation (Wang et al., 2004; Zhao et al., 2020). Accordingly, this species has been listed as a protected species in China since 1988 (Cunningham et al., 2015), as well as Critically Endangered in China (Jiang et al., 2021) and in the International Union for Conservation of Nature and Natural Resources (IUCN) Red List of Threatened Species (IUCN, 2022). To rebuild wild populations, a total of 287,840 captive bred CGS individuals have been released to the wild by the end of October 2019 in China (Shu et al., 2021). However, it is still hard to detect CGS in the wild (except several natural researves; Shu et al., 2021). Therefore, it is urgent to evaluate the health of released CGS, and their microbiota was considered to be a good predictor because of its important functional contribution to host (Gould et al., 2018; Raymann and Moran, 2018). Previous studies have shown that the gastrointestinal microbiota of captive bred CGS can be significantly different in terms of composition and diversity between age groups (Zhang et al., 2018), as well as between groups under different acclimation temperatures (Zhu et al., 2021). However, we still need empirical evidences to evaluate the reorganization of captive bred CGS’s symbiotic bacterial communities when they were released to the wild.

In the present study, the captive bred and the released CGS individuals with different developmental stages and sexes were selected to explore: 1) the sex bias in symbiotic microbiota (skin, oral, stomach, small intestine, and rectum microbiome), 2) the stages bias in symbiotic microbiota, and 3) the differences in symbiotic microbiota between captive bred and released individuals.

## Materials and methods

### Study area

A montane stream located at Dujiangyan, Sichuan Province, China (103°38′22′′–103°39′03′′E, 31°02′18′′–31°02′32′′N) was selected as the study site to conduct the reintroduction activity. This stream has been proved to be suitable for the living of released CGS (Liu, 2021; Zhao et al., 2022). Specifically, this area belongs to a subtropical climate, with the annual precipitation of 1,244 mm, and the average annual temperature of 15.2°C (Liang et al., 2004). The altitude of this stream is approximately 950 m, with deciduous broad-leaved forests and bamboo forests distributing along the riverbank. Chemical oxygen demand and biochemical oxygen demand in the water are extremely low, with little pollution and weak alkaline pH (Liu, 2021). Deep pools with quantitative fish, crabs, and shrimp can be observed in this stream, providing sufficient food resources for reintroduced CGS individuals. More importantly, this area belongs to a private bamboo planting company, ensuring no fishing pressure.

### Study animals

A total of 42 captive bred CGSs belonging to different developmental stages (ages) and sex groups were collected from a hatchery in Hongya County of Sichuan Province, China (103°09′15′′E, 29°52′23′′N). Based on the molecular analyses, they belonged to the Shaanxi clade. These individuals were the offspring of the same parents and were bred in hatchery in 2016 and 2018, respectively. Specifically, 12 individuals including 4 males (age 4), 4 females (age 4), and 4 juveniles (age 2) were considered as the captive bred group. The rest 10 males (age 4), 10 females (age 4), and 10 juveniles (age 2) were used for reintroduction, which were considered as the released group. Before release, these individuals were injected with passive integrated transponder devices (HONGTENG, GuangZhou, China) for individual identification following Gibbons and Andrews (2004). Finally, these individuals were released at six sites (i.e., two sites for upstream, midstream, and downstream, respectively) of the study stream on 22^th^ May, 2020, with each site containing several males, females, and juveniles (Supplementary Figure S1). The released individuals can move freely in this stream to look for their preferred habitats.

### Salamanders recapture and sample collection

We used a combination of visual encounter approach and wire-mesh baited traps (length × width × height = 110 × 60 × 30 cm, mesh size = 8 mm) to recapture the released individuals from 24th June to 19th July, 2020. The visual encounter approach consisted of wading, nocturnal spotlighting, turning substrate, and netting in the stream (Liu et al., 2021). We searched for CGSs in the whole stream after sunset (from 20:00 to 24:00), and individuals encountered were captured by hand nets. Wire-mesh baited traps have been proved to be effective for surveying Cryptobranchids species (Browne et al., 2011). Fresh chicken giblets or frozen hairtail were selected as the baits in a hanging net bag, ensuring CGSs cannot swallow the baits. Traps were placed at the deep pools of the stream at 18:00 every day, and were checked at 6:30 am the next morning. The captured CGSs were carefully transferred on a piece of white nylon cloth one by one by hands wearing sterile gloves. Before microbiota sampling, each individual was rinsed three times by ultra-pure water to remove the potential environmental transient bacteria (Lauer et al., 2007). After that, sterile swabs were immediately used to collect the skin microbes by wiping the dorsal, ventral, and lateral sides of the salamanders (Xu et al., 2020). New sterile swabs were used to gently swab salamanders oral cavity for sampling oral microbiota. The above swabs were preserved into 2 ml sterile centrifuge tubes with 95% alcohol, separately. After that, individuals were euthanized with MS-222 (Webb et al., 2005). They were subsequently dissected to collect the stomach, small intestine, and rectum contents, which were preserved into 2 ml sterile centrifuge tubes, respectively (Zhang et al., 2018). We recaptured 8 juveniles, 6 females and 5 males in total, and all of their microbe samples were used for further analyses. Environmental microbiota were sampled as follows: 5 L of the water in each release site was collected and filtered through 0.45-um micropore membrane (Shi et al., 2013), which were preserved into 2 ml sterile centrifuge tubes with 95% alcohol. Three repetitions were conducted in each release site. All the above samples were preserved into liquid nitrogen in the field, and were then transferred to the laboratory immediately for further analyses. The same sampling processes were conducted for captive bred individuals in the hatchery directly.

### DNA extraction and 16SrRNA amplicon sequencing

All microbiota samples were thawed on ice, and genomic DNA was extracted using a QIAamp Fast DNA Stool Mini Kit (QIAGEN, Hilden, Germany) according to the manufacturer’s protocol, and included a negative extraction control. The quality and quantity of the DNA was verified using 1.0% agarose gel electrophoresis and a NanoDrop spectrophotometer, separately. The V4-V5 region of the 16S rRNA gene was amplified from genomic DNA using 515F (5’-GTGCCAGCMGCCGCGGTAA-3′) and 907R (5’-CCGTCAATTCMTTTGAGTTT-3′) primers (Biddle et al., 2008). The PCR amplification conditions were as follows: initial denaturation 95°C for 5 min, followed by 35 cycles of 95°C for 30 s, 55°C for 30 s, and 72°C for 45 s, with a final extension step at 72°C for 10 min. High-throughput sequencing of barcoded amplicons was performed using the Illumina MiSeq platform by Mingke Biotechnology Co., Ltd. (Hangzhou, China).

### Microbiota sequence analyses

We used QIIME 1.9 to process the raw reads and to obtain clean sequences (Caporaso et al., 2010). The *search*, *flash*, and *trimmomatic* function were used for removing low-quality sequences, splicing and quality control, respectively (Edgar, 2010). Operational taxonomic units (OTUs) were defined as the identity sharing >97% sequences, and representative sequences (the largest number of sequences in each OTU) were classified against the SILVA132 database (Quast et al., 2013). Finally, we obtained OTU abundance tables containing taxon information. To standardize the number of reads across samples for our main analyses, all samples were rarefied to 26,484 sequences (the lowest number of sequences of all samples in this study).

### Statistical analyses

Alpha diversity (i.e., observed OTUs and Shannon index) was calculated for each sample in QIIME 1.9 according to the relative abundance-based OTU table (Caporaso et al., 2010). All alpha diversity values were assessed for normality using Shapiro–Wilk tests (Varona et al., 2005). Alpha diversity indices with normal distribution were compared using Student’s t-test, while Mann–Whitney U test was conducted to compare indices with non-normal distribution. Beta diversity was calculated with Bray-Curtis, unweighted UniFrac, and weighted UniFrac dissimilarity metrics by QIIME pipeline, respectively. PERMANOVA was performed to determine the difference of microbiome composition between groups at OTU level, and principal coordinates analysis (PCoA, based on dissimilarity matrices) was used to visualize the dissimilarity of beta diversity. Benjamini–Hochberg (BH) correction was used to obtain the corrected *p*-values. We evaluated the variation of symbiotic microbial composition between captive bred and released individuals using the top 10 families and genera based on the Mann–Whitney U analysis. The linear discriminant analysis effect size (LEfSe) was performed to explore the differentially-abundant bacterial taxa between groups (Segata et al., 2011). Source-Tracker 0.9.5 was used to calculate the relative contribution of habitat and original symbiotic microbiota to the reorganization of released individuals microbiome (Knights et al., 2011). LEfSe analyses were performed on the Galaxy web-based platform.^1^ Other analyses were conducted in R (R Core Team, 2020). Specifically, the Shapiro–Wilk test was performed using the *stats* package (R Core Team, 2020). Student’s t-test and Mann–Whitney U test were conducted using *ggpubr* package (Whitehead et al., 2019). PERMANOVA was performed using the *vegan* package (Dixon, 2003). PCoA was conducted using *ape* package (Paradis et al., 2004). And figures were created using *ggplot2* package (Ginestet, 2011).

## Result

### Sex bias in symbiotic microbiota

**Figure 1:**
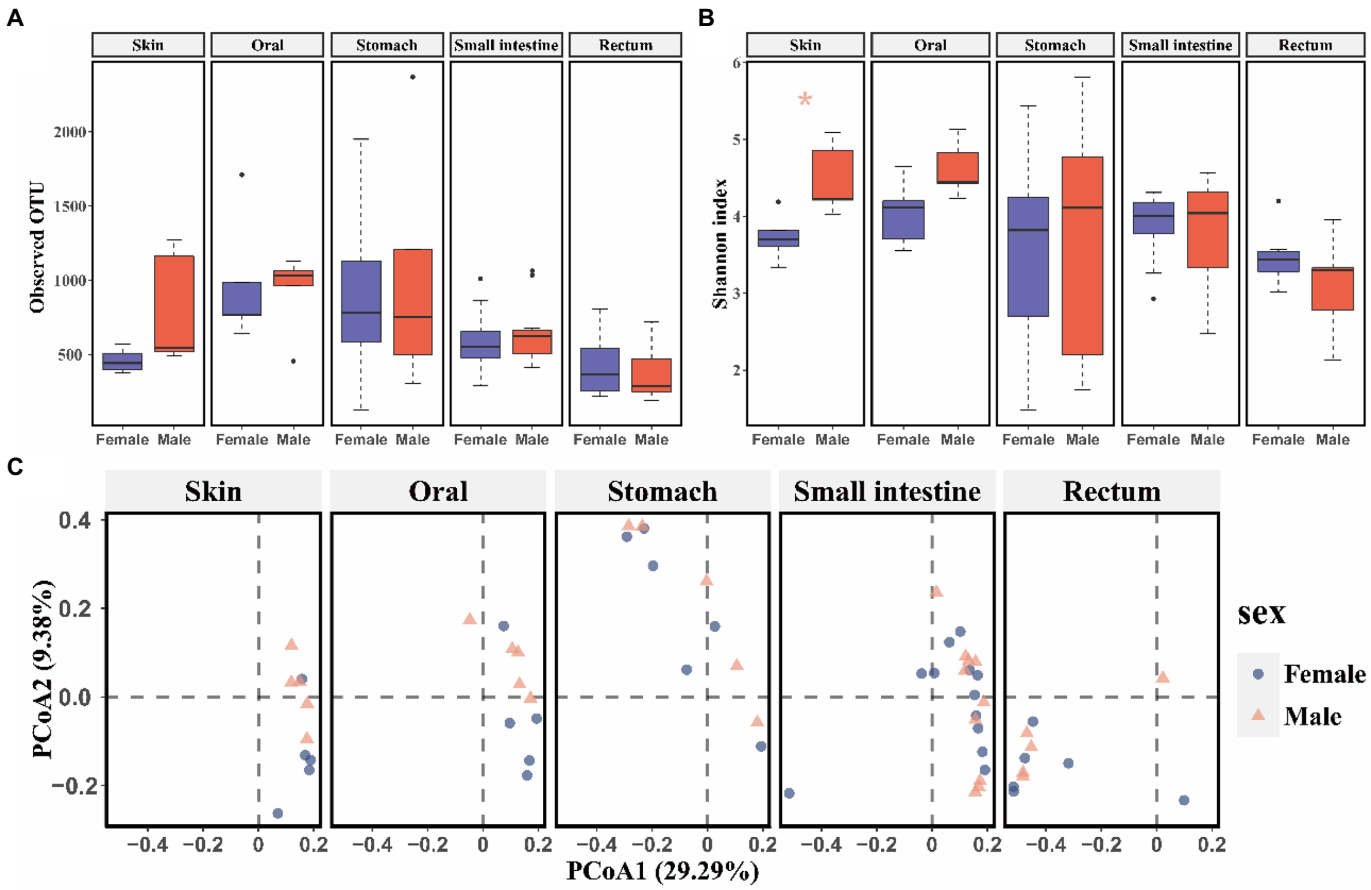
The comparison of alpha and beta diversity of symbiotic microbiota between released female and male CGSs. **(A)** Boxplot of observed OTU values; **(B)** Boxplot of Shannon diversity values. Data are presented as means ± SE, and significant differences are marked with an asterisk; **(C)** PCoA scatter plots present the dissimilarity of microbiomes at the OTU level based on Bray-Curtis distance.

No significant differences of alpha (*p* > 0.05, Supplementary Table S1) and beta diversity (*p* > 0.05, PERMANOVA and BH correction) were detected in skin, oral, stomach, small intestine, and rectum microbiome between captive bred males and females. However, released males exhibited higher Shannon diversity (t test, *p* = 0.019, Figure 1B) than females in skin bacterial communities. Additionally, beta diversity of the skin bacterial communities differed between released males and females (PERMANOVA and BH correction: *R*^2^ = 0.18, *p* = 0.048 for Bray-Curtis; *R*^2^ = 0.15, *p* = 0.039 for unweighted Unifrac; *R*^2^ = 0.19, *p* = 0.048 for weighted Unifrac metrics; Figure 1C). A LEfSe analysis revealed 19 divergent taxa of skin bacterial communities between sexes (5 for females and 14 for males; α = 0.05，LDA = 3.2, Supplementary Figure S2A). Specifically, released females had higher relative abundance of Bacilli (Females: 45.7%, Males: 34.4%), Lactobacillales (females: 45.4%, males: 33.2%), Lactobacillaceae (females: 44.8%, males: 32.3%) and *Lactobacillus* (females: 44.8%, males: 32.3%) than released males at class, order, family, and genus levels, respectively (Supplementary Figure S2B).

### Stage bias in symbiotic microbes

**Figure 2:**
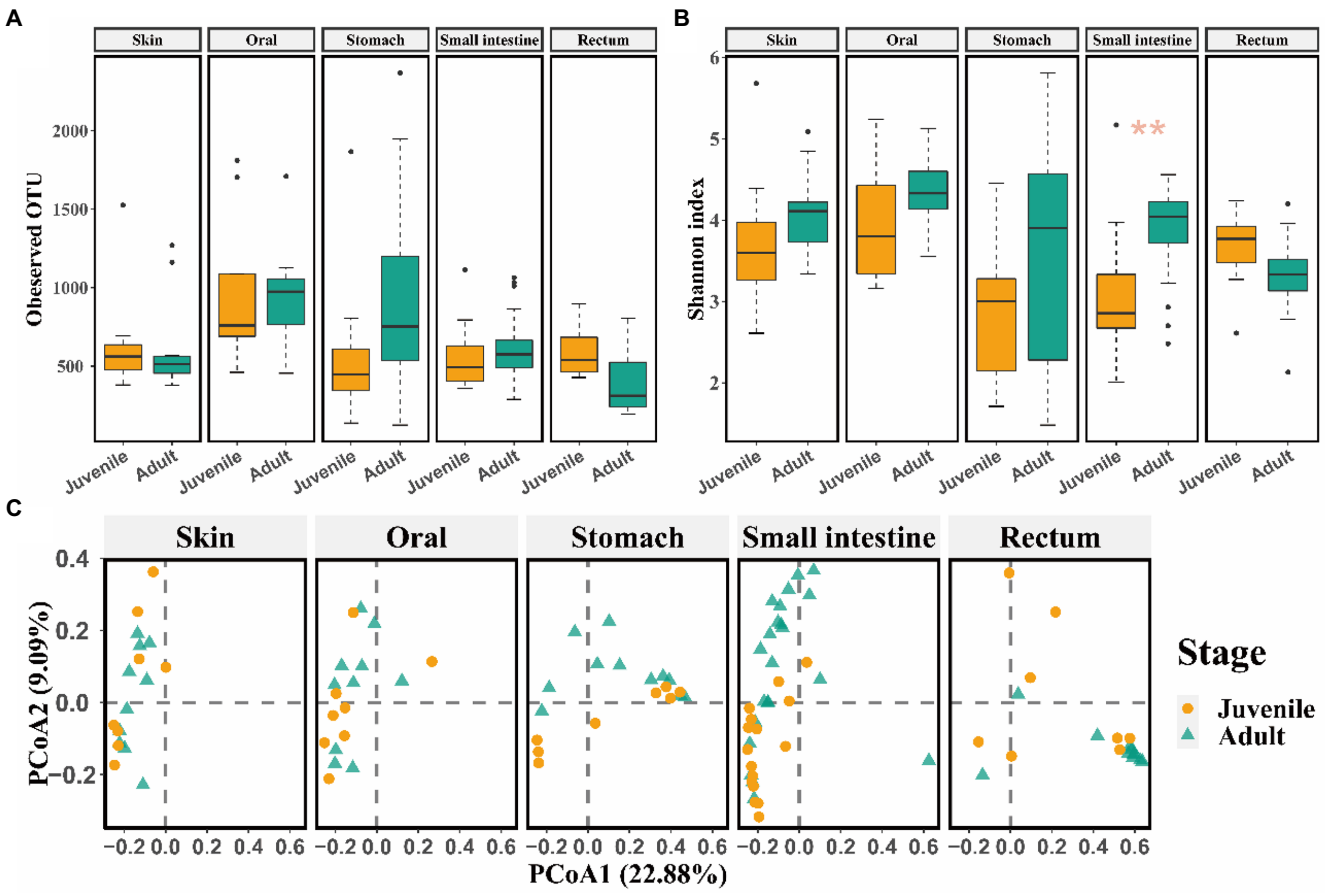
The comparison of alpha and beta diversity of symbiotic microbiota between released juvenile and adult CGSs. **(A)** Boxplot of observed OTU values; **(B)** Boxplot of Shannon diversity values. Data are presented as means ± SE, and significant differences are marked with an asterisk; **(C)** PCoA scatter plots present the dissimilarity of microbiomes at the OTU level based on Bray-Curtis distance.

Captive bred juveniles had higher OTU richness (observed OTU: Mann–Whitney U test, *p* = 0.004) and Shannon diversity (*t* test, *p* = 0.010) in the rectum microbiome, and higher OTU richness (observed OTU: Mann–Whitney U test, *p* = 0.008) in the skin microbiome compared to captive bred adults (Supplementary Figures S3A,B). Moreover, the captive bred juveniles and adults differed in beta diversity of skin and rectum microbiome (rectum: *R*^2^ = 0.62, *p* = 0.002 for Bray-Curtis; *R*^2^ = 0.29, *p* = 0.002 for unweighted Unifrac; *R*^2^ = 0.58, *p* = 0.002 for weighted Unifrac metrics; PERMANOVA and BH correction; skin: *R*^2^ = 0.74, *p* = 0.002 for Bray-Curtis; *R*^2^ = 0.39, *p* = 0.002 for unweighted Unifrac; *R*^2^ = 0.79, *p* = 0.002 for weighted Unifrac metrics; PERMANOVA and BH correction; Supplementary Figure S3C). In contrast, released adults have higher Shannon diversity (t test, *p* = 0.002) in the small intestine microbiota compared to released juveniles (Figures 2A,B). In addition, released adults and juveniles also differed significantly in beta diversity of the small intestine microbiota (*R*^2^ = 0.11, *p* < 0.001 for Bray-Curtis; *R*^2^ = 0.04, *p* = 0.009 for unweighted Unifrac; *R*^2^ = 0.10, *p* < 0.001 for weighted Unifrac metrics; PERMANOVA and BH correction). The bacterial communities of small intestine can be clustered into two groups by the PCoA plot according to the developmental stages (i.e., juveniles and adults; Figure 2C). Based on the LEfSe analyses, released juveniles had higher relative abundance of Firmicutes at phylum level, while Bacteroidetes, Bacteroidia, Bacteroidales, S24-7, and unclassified genus of S24-7 were more abundant in released adults at phylum, class, order, family, and genus levels, respectively (Supplementary Figures S4A,B).

### Differences of microbial symbionts between captive bred and released individuals

No significant difference of alpha diversity and beta diversity was detected in skin, oral, stomach, small intestine, and rectum microbiome between captive bred males and females (*p* > 0.050). And marginal difference was only observed in skin microbiota composition between released females and males (*p* = 0.048 for Bray-Curtis and weighted Unifrac, *p* = 0.039 for unweighted Unifrac metrics). Therefore, females and males were pooled together (i.e., released adults) to explore the differences in microbial symbionts between captive bred and released adults.

**Figure 3:**
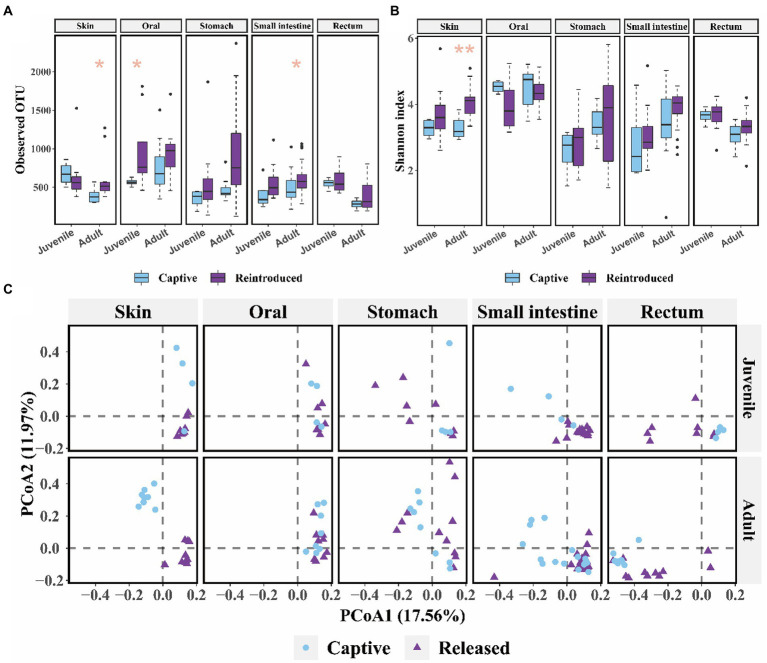
The comparison of alpha and beta diversity between captive bred and released CGSs symbiotic microbiota. **(A)** Boxplot of observed OTU values; **(B)** Boxplot of Shannon diversity values. Data are presented as means ± SE, and significant differences are marked with an asterisk; **(C)** PCoA scatter plots present the dissimilarity of microbiome at the OTU level, based on weighted Unifrac distance.

Released adults had significant higher alpha diversity (observed OTU: Mann–Whitney U test, *p* = 0.021; Shannon: t test, *p* = 0.002) in the skin microbiome and more diverse OTU richness (observed OTU: Mann–Whitney U test, *p* = 0.048) in the small intestine bacterial community than captive bred adults (Figures 3A,B). And released juveniles had significant higher oral bacteria OTU richness (observed OTU: Mann–Whitney U test, *p* = 0.048, Figure 3A) compared to captive bred juveniles. There was a significant difference in the skin, small intestine, and rectum bacterial composition between captive bred and released juveniles, as well as that between captive bred and released adults (*p* < 0.050, PEMANOVA and BH correction). These microbiota samples could be divided into captive bred and released groups by PCoA plots (Figure 3C).

**Figure 4:**
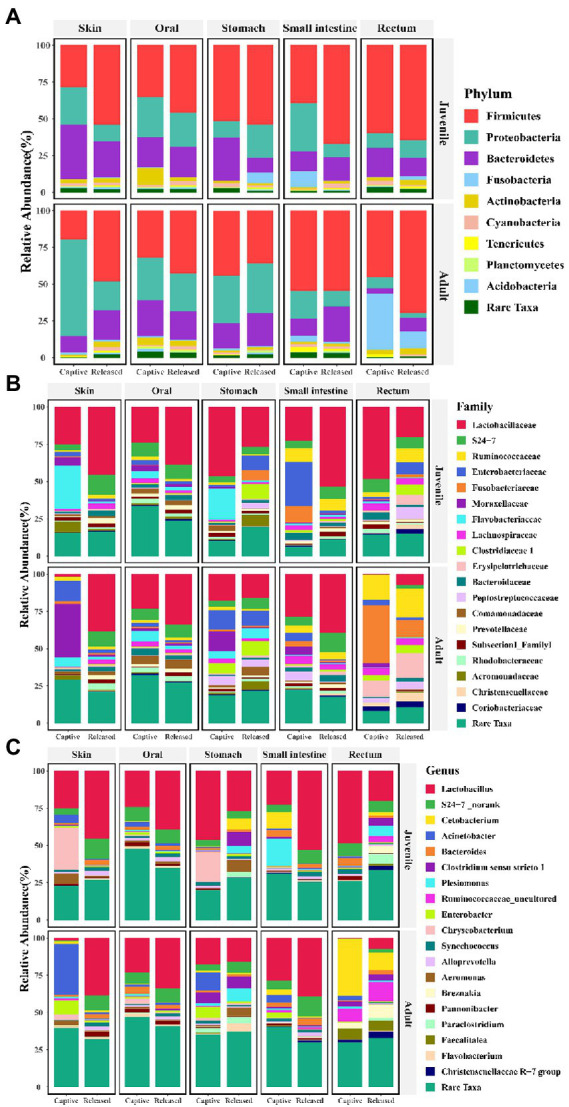
Bar plots show the symbiotic microbiota composition between captive bred and released individuals at phylum **(A)**, family **(B)**, and genus **(C)** level.

**Figure 5:**
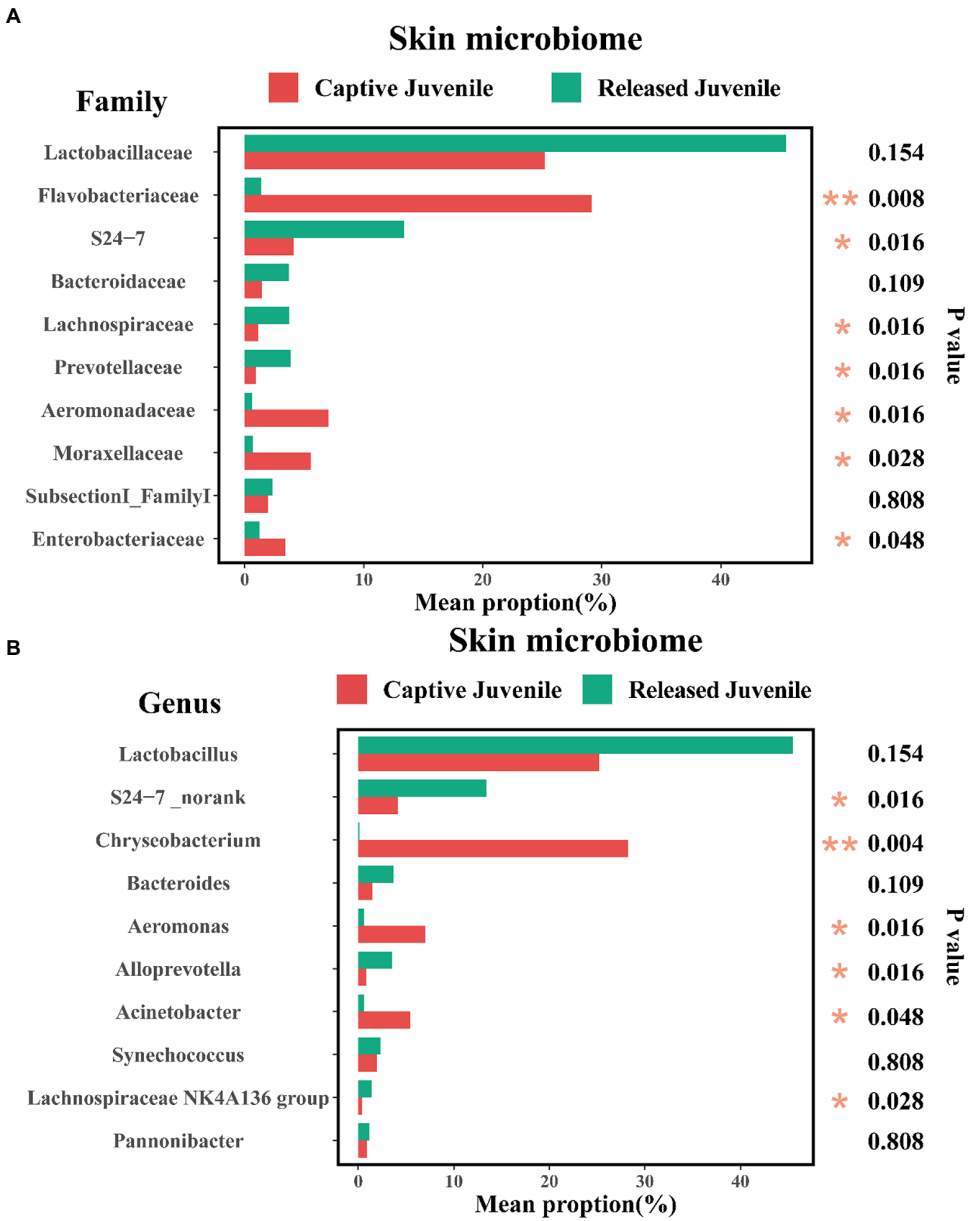
Variations of top 10 families **(A)**, and genera **(B)**, of skin microbiome between captive bred and released juveniles. A Mann–Whitney U test was used to evaluate the variation across two groups. Significance was set at the 0.05 level.

The community composition of symbiotic microbiome was showed in Figures 4A–C at phylum, family, and genus level, respectively. Seven known taxa of the top 10 families and six known taxa of the top 10 genera in skin microbiome showed significant variation between captive bred and released juveniles (Mann–Whitney U test, *p* < 0.05; Figures 5A,B). Specifically, the predominant family S24-7 and unclassified genus of S24-7 were more abundant in released juveniles, whereas the predominant family Flavobacteriaceae and genus *Chryseobacterium* were more abundant in captive bred juveniles. Nine known taxa of the top 10 families and 10 known taxa of the top 10 genera were significantly different between captive bred and released adults (Mann–Whitney U test, *p* < 0.05; Supplementary Tables S2 and S3). Specifically, the predominant family Lactobacillaceae and genus *Lactobacillus* were more abundant in released adults, whereas the predominant family Moraxellaceae and genus *Acinetobacter* were more abundant in captive bred adults.

In the small intestine microbiome, we found that four known taxa of the top 10 families and seven known taxa of the top 10 genera showed significant variation between captive bred and released juveniles (Mann–Whitney U test, *p* < 0.05; Supplementary Tables S4 and S5). Specifically, the predominant family Lactobacillaceae and genus *Lactobacillus* were more abundant in released juveniles, whereas the predominant genus *Plesiomonas* were more abundant in captive bred juveniles. Six known taxa of the top 10 families and seven known taxa of the top 10 genera were significantly different between captive bred and released adults (Mann–Whitney U test, *p* < 0.05; Supplementary Tables S6 and S7). Specifically, the predominant family S24-7 and unclassified genus of S24-7, genus Bacteroides were more abundant in released adults.

In the rectum microbiome, we found that four known taxa of the top 10 families and three known taxa of the top 10 genera showed significant variation between captive bred and released juveniles (Mann–Whitney U test, *p* < 0.05; Supplementary Tables S8 and S9). Specifically, the predominant family Lactobacillaceae and genus *Lactobacillus* were more abundant in captive bred juveniles. Only one known taxa of the top 10 families and one known taxa of the top 10genera were significantly different between captive bred and released adults (Mann–Whitney U test, *p* < 0.05; Supplementary Tables S10 and S11). Specifically, the predominant family Fusobacteriaceae and genus *Cetobacterium* were more abundant in captive bred adults.

**Figure 6:**
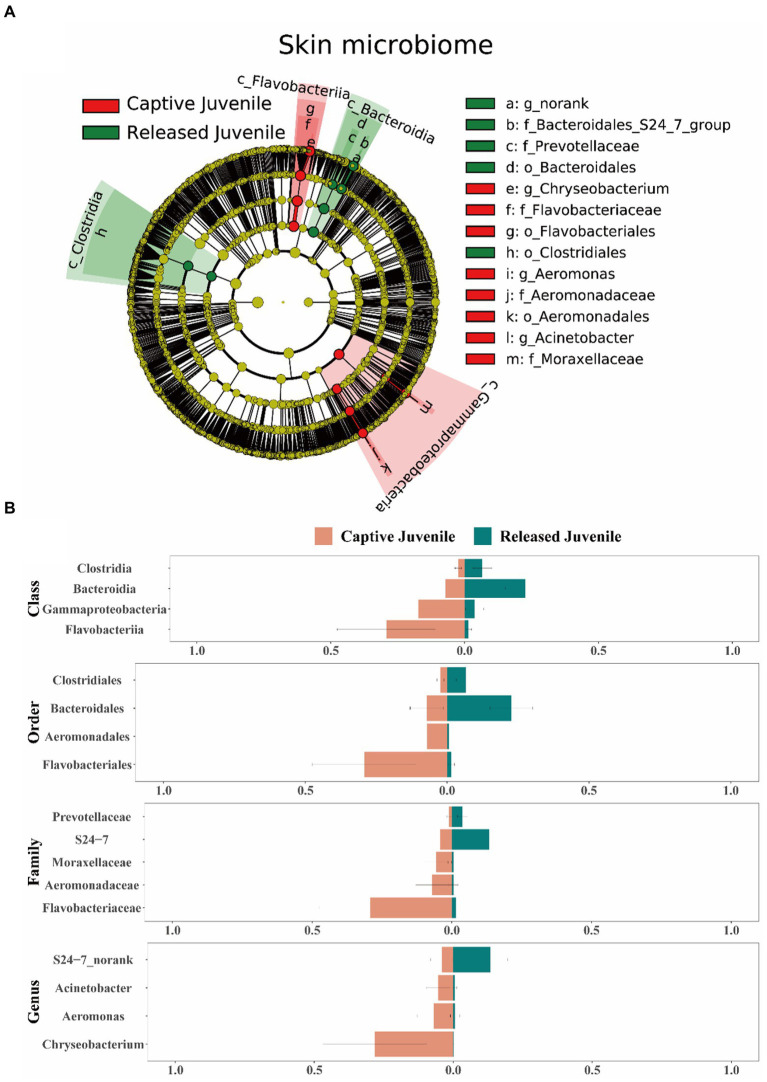
Difference of the skin microbiota between captive bred and released juveniles. **(A)** A LEfSe analysis identifies the different abundant skin bacterial taxa between captive bred and released juveniles. **(B)** Side-by-side comparison of the mean relative abundance of different abundant skin bacterial taxa between captive bred and released juveniles at class, order, family, and genus levels. Data are presented as means ± SE in bar graphs.

In terms of the skin microbiome, LEfSe analysis indicated that Bacteroidia, Bacteroidales, S24-7, and unclassified genus of S24-7 were more abundant in released juveniles, whereas Flavobacteriia, Flavobacteriales, Flavobacteriaceae, and *Chryseobacterium* were more abundant in captive bred juveniles at class, order, family, and genus levels, respectively (Figures 6A,B). Released adults had higher relative abundance of Firmicutes, Bacilli, Lactobacillales, Lactobacillaceae, and *Lactobacillus*, while captive bred adults had higher relative abundance of Proteobacteria, Gammaproteobacteria, Pseudomonadales, Moraxellaceae, and *Acinetobacter* at phylum, class, order, family, and genus levels, respectively (Supplementary Figures S5A,B). In the small intestine microbiome, released juveniles had higher relative abundance of Firmicutes, Bacilli, Lactobacillales, Lactobacillaceae, and *Lactobacillus*, while captive bred juveniles had higher relative abundance of Proteobacteria, Gammaproteobacteria, Enterobacteriales, Enterobacteriaceae, and *Plesiomonas* at phylum, class, order, family, and genus levels, respectively (Supplementary Figures S6A,B). Bacteroidetes, Bacteroidia, Bacteroidales, S24-7, and unclassified genus of S24-7 were more abundant in released adults compared to captive bred adults at phylum, class, order, family, and genus levels, respectively (Supplementary Figures S7A,B). In the rectum microbiome, Clostridia, Clostridiales, Clostridiaceae_1, and *Clostridium_sensu_stricto_1* were more abundant in released juveniles, whereas Bacilli, Lactobacillales, Lactobacillaceae, and *Lactobacillus* were more abundant in captive bred juveniles at class, order, family, and genus levels, respectively (Supplementary Figures S8A,B). In addition, released adults had higher relative abundance of Firmicutes at phylum level, while captive bred adults had higher relative abundance of Fusobacteriia, Fusobacteriales, Fusobacteriaceae, and *Cetobacterium* at class, order, family, and genus levels, respectively (Supplementary Figures S9A,B).

Differences of water environment microbiome were also observed between hatchery and the released sites (Supplementary Figures S10A,B). At the phylum level, Proteobacteria were more abundant in captive bred environment, whereas Firmicutes were more abundant in released environment (Supplementary Figure S10A). At the genus level, *Chryseobacterium* and unclassified genus of Comamonadaceae were more abundant in captive bred environment, while *Lactobacillus* and unclassified genus of S24-7 were more abundant in released environment (Supplementary Figure S10B). In addition, source-track analyses indicated that environmental microbiome were the major sources of skin microbiome for both released females (67.69%) and males (57.84%; Supplementary Figure S11).

## Discussion

### Sex bias in symbiotic microbiota

No significant differences of alpha and beta diversity were observed in all the symbiotic microbiome between captive bred males and females. This probably because all captive bred adults were raised under similar external environmental conditions (e.g., the water supply, food, and temperature) in the hatchery. However, this situation has been strongly changed in the skin microbial community between released females and males, as they exhibited significant difference in alpha and beta diversity. A previous study demonstrated that the linear home range and daily movement of released male CGSs were significantly higher (i.e., more diverse habitat utilization) than those of released females (Zhao et al., 2022). Therefore, such skin microbial community differences between sexes should be attributed to males and females different microhabitat utilization in the field, as amphibian skin microorganisms are susceptible to environmental factors (Loudon et al., 2014; Muletz Wolz et al., 2018; Varela et al., 2018; Hernandez-Gomez et al., 2019). This can be also supported by our results from the source-track analyses showing that environmental microbiota was the main sources of released individuals skin microbiome. More importantly, amphibian skin is an important immune organ (Varga et al., 2018), the higher diversity of skin bacterial communities may better protect amphibians against pathogens (Piovia-Scott et al., 2017). Therefore, this could be one of the reasons that the survival rate of released male CGSs was higher than released females in the studied stream (Liu, 2021).

### Stage bias in symbiotic microbes

The skin and rectum bacterial community between captive bred juveniles and adults were strongly divergent. Specifically, captive bred juveniles had higher alpha diversity in the rectum microbiome, and higher OTU richness in the skin microbiome compared to captive bred adults. This is in contrast with a previous study that indicated the alpha diversity of gastrointestinal microbial community in captive bred adults was higher than captive bred juveniles (Zhang et al., 2018). This is because CGSs were raised under different environment in different hatcheries (e.g., temperature, water condition, and food resources). In the present study, captive bred juveniles and adults are raised under similar environmental conditions except food supply (red worms *Chironomus* sp. and frozen fish *Hemiculter leucisculus* for juveniles while only frozen fish for adults). Previous studies indicated that more diverse food resources can induce higher diversity of skin and gut microbiome in the same amphibian species, such as the red-eyed tree frog (*Agalychnis callidryas*) in Belize (Antwis et al., 2014). Therefore, more diverse diet may contribute to a higher alpha diversity of skin and rectum microbiome in captive bred juveniles than adults. Furthermore, captive bred juveniles had higher relative abundance of *Lactobacillus* in the skin and rectum microbiome than adults. Since their living environment is similar, food items could be the potential important contributors to the colonization of *Lactobacillus*. Source-tracking analyses can be used in future studies to verify this inference.

In terms of the released group, adults and juveniles differed in Shannon and beta diversity of small intestine microbiome. Released adults should be the top predators in the stream ecosystem, consuming a wide range of food items such as fish, craps, and shrimps (Fei et al., 2006). In contrast, released juveniles may concentrated in preying limited food resources as they occupied smaller habitat niche in the stream (Zhao et al., 2022). Accordingly, higher Shannon diversity of small intestine microbiome in released adults may contribute to digest more kinds of food than released juveniles. This can be also supported by the higher relative abundance of S24-7 in released adults, as S24-7 microbes were associated with diverse complex carbohydrate degradation and the breakdown of proteins (Ormerod et al., 2016; Lagkouvardos et al., 2019).

### Differences in microbial symbionts between captive bred and released individuals

Overall, released juveniles and adults had higher alpha diversity in microbiome than captive bred juveniles and adults, respectively. This pattern was consistent with previous studies showing that wild individuals of *Lissotriton vulgaris*, *Triturus cristatus*, and *Cynops pyrrhogaster* exhibited higher alpha diversity of cutaneous bacteria than captive bred individuals (Sabino-Pinto et al., 2016; Bates et al., 2019). Because lower diversity of symbiotic microbiome may lead to a higher susceptibility of hosts to diseases (Becker and Harris, 2010), our results may explain why captive bred CGSs were easily infected by bacteria, fungi, and parasites (Zhou et al., 2012). Accordingly, increasing the alpha diversity of symbiotic microbiome (e.g., the application of microbial inoculum) may be helpful to promote the survival of CGSs. Moreover, the increase of diversity of symbiotic microbiome in released individuals can be attributed to a shift of a simple to complex living environment, which promote their ability to defense against pathogenic bacteria (Antwis et al., 2014; Bataille et al., 2016). This can explain the observations that few infected CGSs were detected in the field (Liu et al., 2021). Accordingly, pre-exposure to the field water conditions of CGSs before reintroduction can be an effective approach to help the colonization of diverse microbes, and thus enhance the survival of released individuals (Zhu et al., 2022). This is especially true for the juveniles, as we found that released juveniles exhibited lower alpha diversity of microbiome than adults. However, more evidences are still needed in future studies.

Microbiome composition was also significantly changed when comparing captive bred individuals with released individuals. Genus *Chryseobacterium* and *Plesiomonas* were more abundant in captive bred juveniles, while genus *Acinetobacter* were more abundant in captive bred adults. Since *Chryseobacterium* was the abundant genus in captive bred environment, our results supported the previous findings that hosts symbiotic microbes were associated with environmental microbes (Muletz Wolz et al., 2018; Moeller et al., 2020). However, symbiotic microbes were also affected by food resources (Antwis et al., 2014; Chang et al., 2016; Edwards et al., 2017). This may be the reason that abundant genus were different between captive bred juveniles and adults, which were provided different food in the hatchery. In contrast, the abundant genera were changed into *Lactobacillus* and unclassified genus of S24-7 in both released juveniles and adults. These genera contributed a lot to the released environmental microbes, which play more important roles to determine released individuals symbiotic microbes. Future studies can investigate using environmental microbes to infer hosts health in the field.

## Conclusion

Our results indicated no sex but stage bias of symbiotic microbiome in captive bred CGSs. However, this intraspecific variation patterns of symbiotic microbiome can be modified when captive bred individuals were released to the field. Overall, we observed a lower alpha diversity and higher relative abundance of *Chryseobacterium*, *Plesiomonas*, and *Acinetobacter* in the bacterial community of captive bred individuals. Instead, higher alpha diversity of symbiotic microbiota and higher relative abundance of S24-7 and *Lactobacillus* was detected in released individuals. Whether these modifications are related to specific functions for the adaptation of released CGSs could be tested. Moreover, since the effectiveness of CGS reintroduction is limited in most of the freshwater ecosystems, future studies can incorporate other approaches (e.g., blood physiology) to better evaluate the growth and health of reintroduced CGSs.

## Data availability statement

The datasets presented in this study can be found in online repositories. The names of the repository/repositories and accession number(s) can be found at: https://ngdc.cncb.ac.cn/gsub/submit/gsa/subSAM102547, PRJCA012924.

## Ethics statement

The animal study was reviewed and approved by the Animal Ethical and Welfare Committee of Chengdu Institute of Biology, Chinese Academy of Sciences.

## Author contributions

TZ, WZ, and JF conceived this study. JF, CZ, TZ, and ZS collected the samples and performed the experiments. JF and WZ analyzed the data. JF and TZ wrote the manuscript. All authors contributed to the article and approved the submitted version.

## Funding

This work was supported by the National Key Programme of Research and Development, Ministry of Science and Technology (2016YFC0503200), the Biodiversity Survey and Assessment Project of the Ministry of Ecology and Environment, China (2019HJ2096001006), Construction of Basic Conditions Platform of Sichuan Science and Technology Department (2019JDPT0020), the opening project of Hunan Engineering Laboratory for Chinese Giant Salamander’s Resource Protection and Comprehensive Utilization in Jishou University (DNGC2201), and China Biodiversity Observation Networks (Sino BON).

## Conflict of interest

The authors declare that the research was conducted in the absence of any commercial or financial relationships that could be construed as a potential conflict of interest.

## Publisher’s note

All claims expressed in this article are solely those of the authors and do not necessarily represent those of their affiliated organizations, or those of the publisher, the editors and the reviewers. Any product that may be evaluated in this article, or claim that may be made by its manufacturer, is not guaranteed or endorsed by the publisher.

## Footnotes

^1^ http://huttenhower.sph.harvard.edu/galaxy/
