# Supplementary material for: Reintroduction modifies the intraspecific variations of symbiotic microbes in captive bred Chinese giant salamander
Source: Front Microbiol. 2022 Dec 1;13:1062604. doi: 10.3389/fmicb.2022.1062604 (PMC9751345; doi:10.3389/fmicb.2022.1062604)
Supplement: Supplementary file 1 [file Data_Sheet_1.docx]

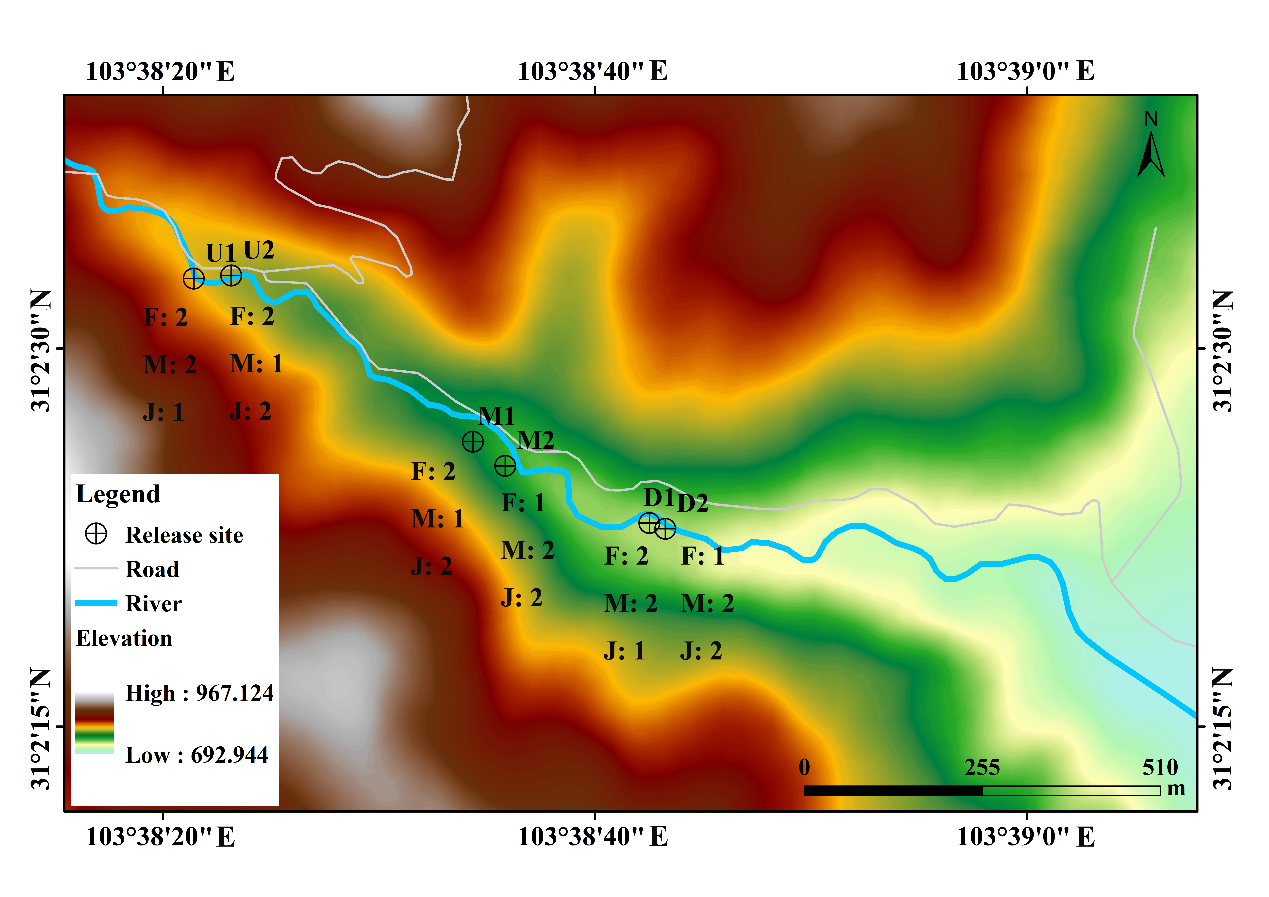


**Figure S1 |** Geographical location of the study area and the released number of CGSs (F: females, M: males and J: juveniles) at each released site.


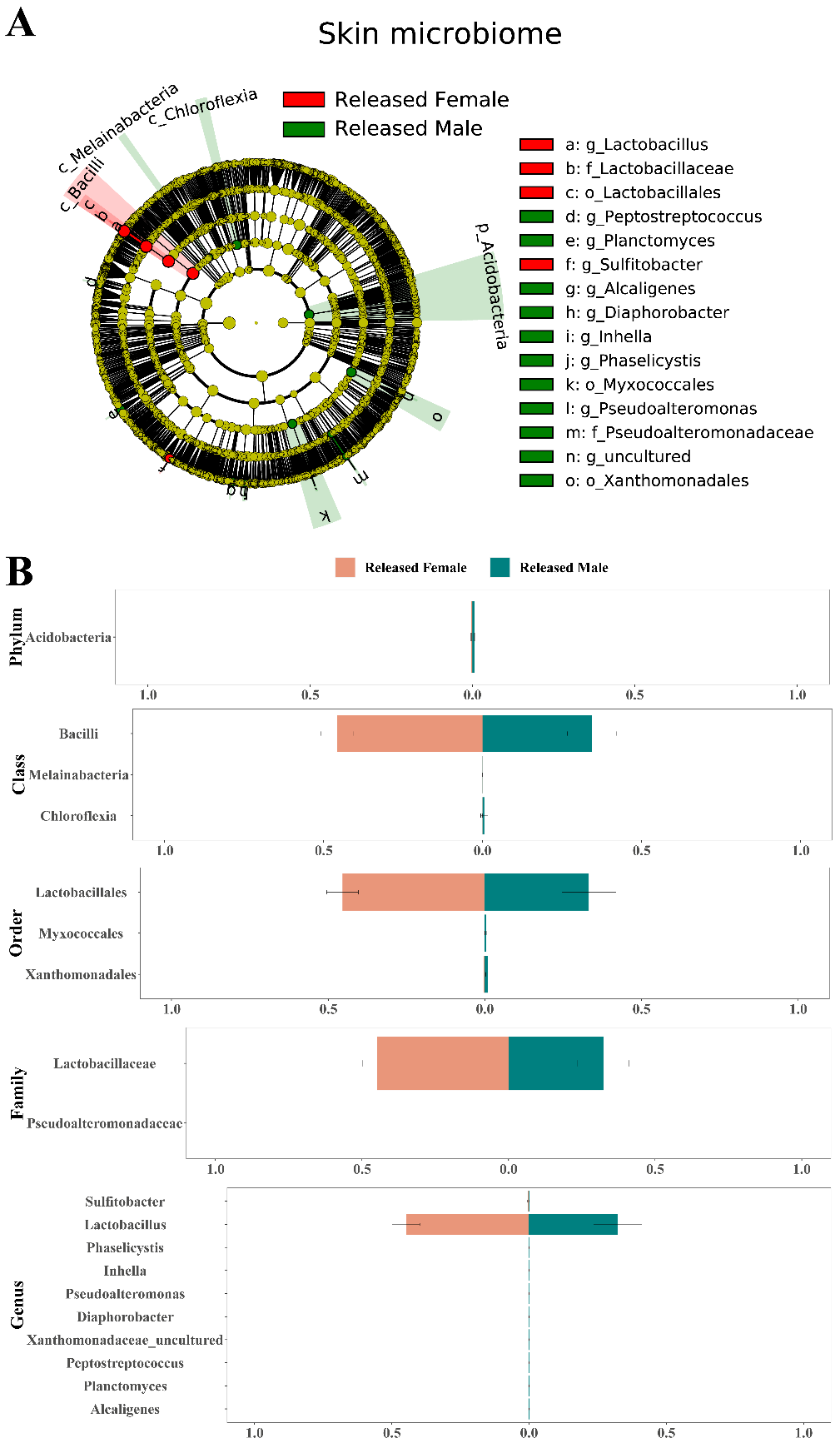


**Figure S2 |** Difference of the skin microbiota between released females and males. **(A)** A LEfSe analysis identifies the different abundant skin bacterial taxa between released females and males. **(B)** Side-by-side comparison of the mean relative abundance of different abundant skin bacterial taxa between released females and males at phylum, class, order, family, and genus levels. Data are presented as means ± SE in bar graphs.


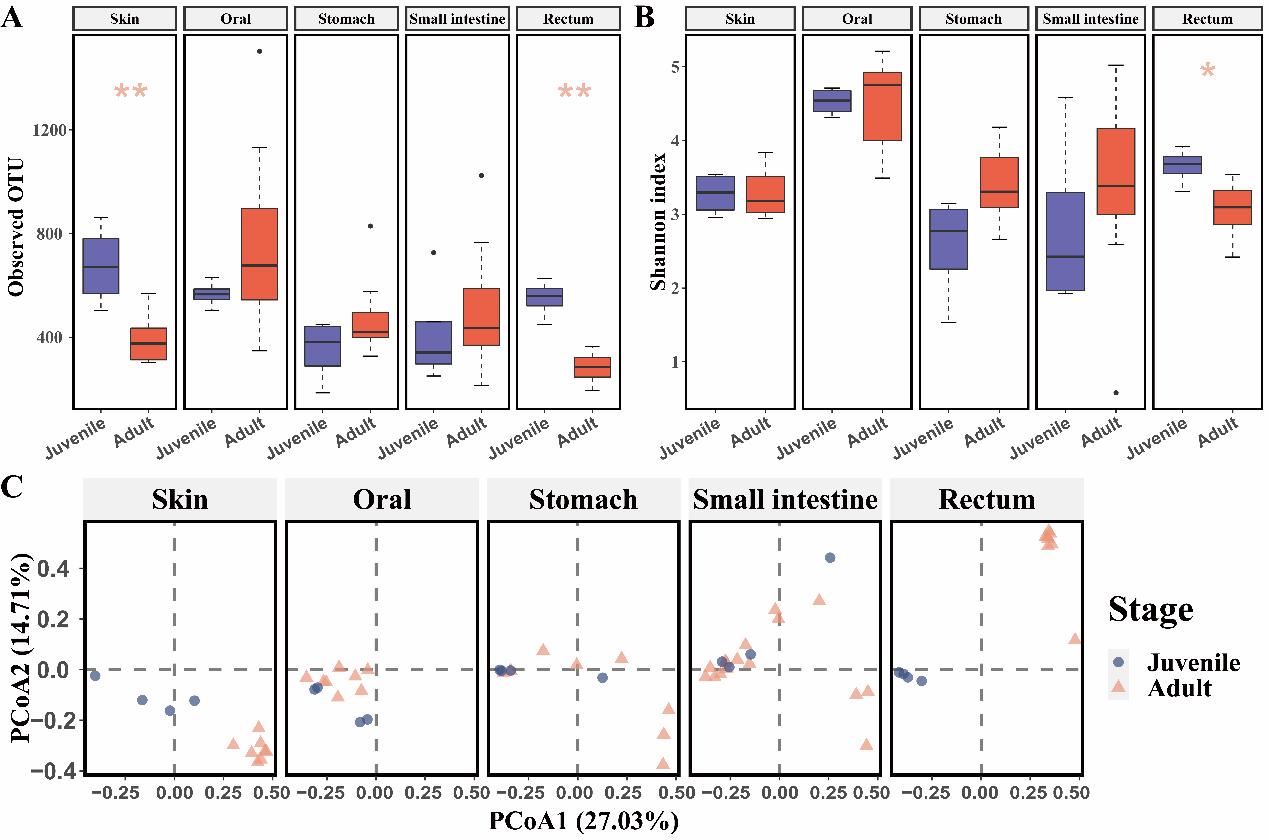


**Figure S3 |** The comparisons of alpha and beta diversity between captive bred juvenile and adult CGSs symbiotic microbiota. **(A)** Boxplot of the observed OTU values; **(B)** Boxplot of the Shannon diversity values. Data are presented as means ± SE and significant differences are marked with an asterisk; **(C)** PCoA scatter plots present the dissimilarity of microbiomes at the OTU level based on the Bray-Curtis distance.


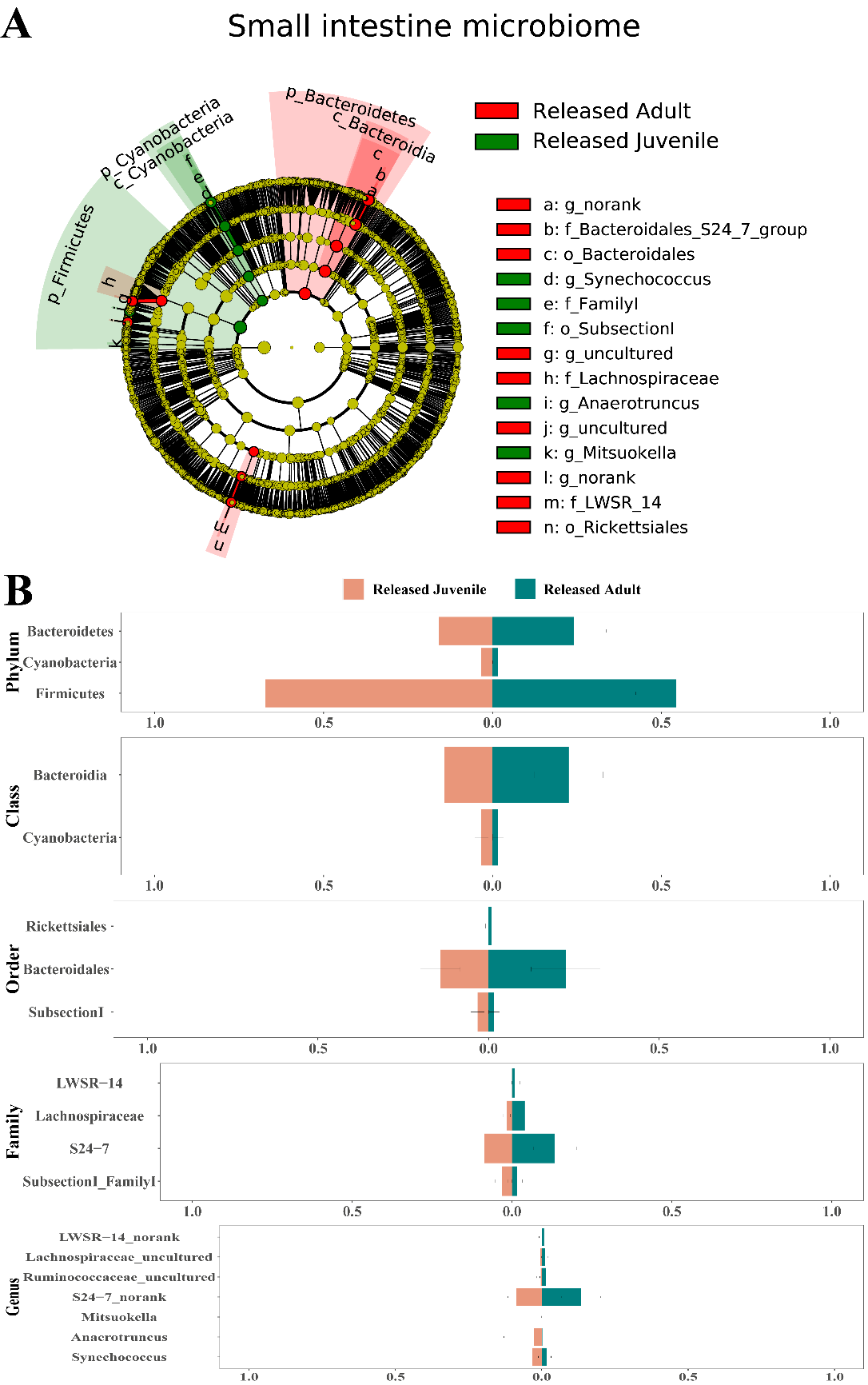


**Figure S4 |** Difference of the small intestine microbiota between released juveniles and adults. **(A)** A LEfSe analysis identifies the different abundant small intestine bacterial taxa between released juveniles and adults. **(B)** Side-by-side comparison of the mean relative abundance of different abundant small intestine bacterial taxa between released juveniles and adults at phylum, class, order, family, and genus levels. Data are presented as means ± SE in bar graphs.


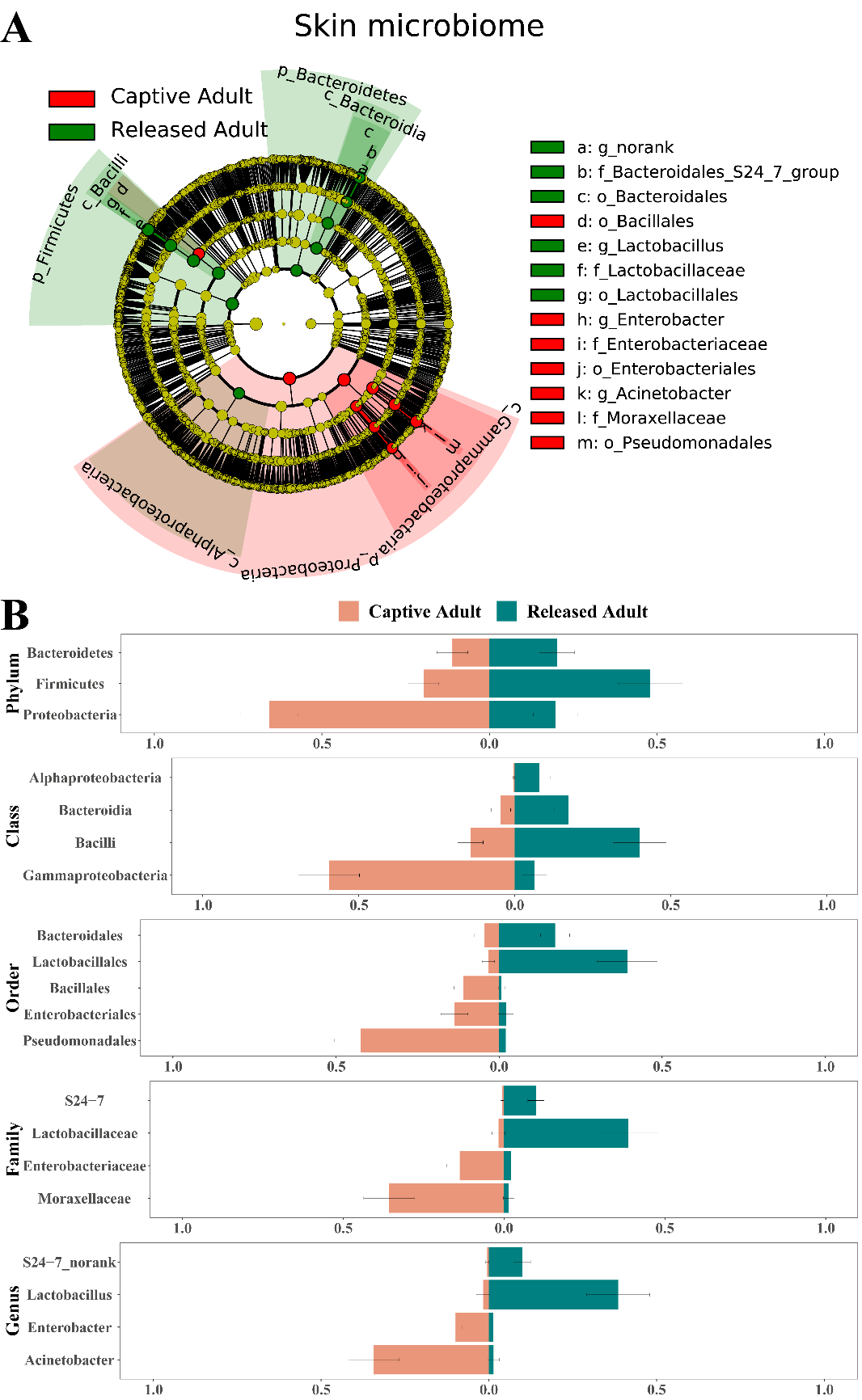


**Figure S5 |** Difference of the skin microbiota between captive bred and released adults. **(A)** A LEfSe analysis identifies the different abundant skin bacterial taxa between captive bred and released adults. **(B)** Side-by-side comparison of the mean relative abundance of different abundant skin bacterial taxa between captive bred and released adults at phylum, class, order, family, and genus levels. Data are presented as means ± SE in bar graphs.


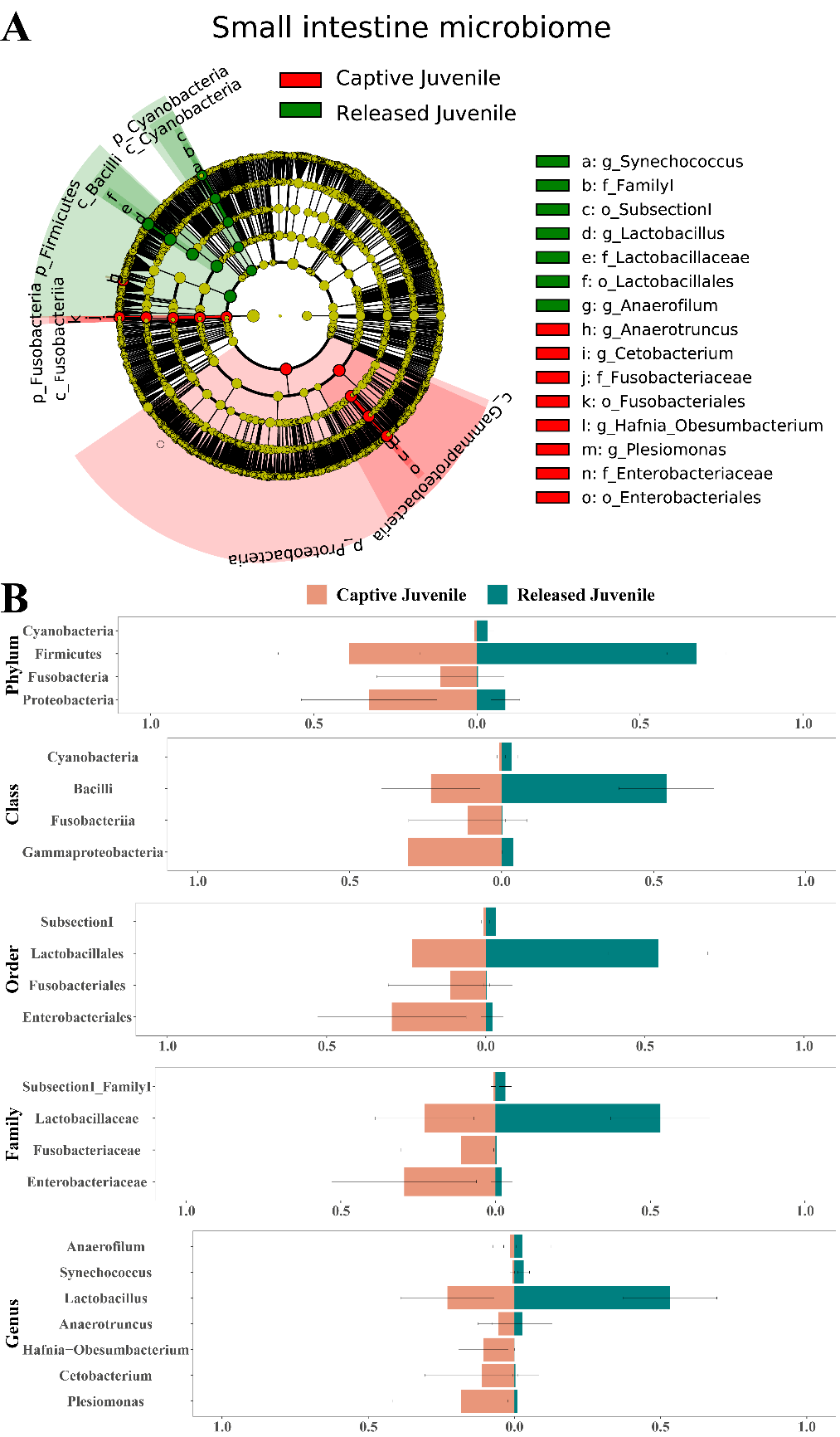


**Figure S6 |** Difference of the small intestine microbiota between captive bred and released juveniles. **(A)** A LEfSe analysis identifies the different abundant small intestine bacterial taxa between captive bred and released juveniles. **(B)** Side-by-side comparison of the mean relative abundance of different abundant small intestine bacterial taxa between captive bred and released juveniles at phylum, class, order, family, and genus levels. Data are presented as means ± SE in bar graphs.


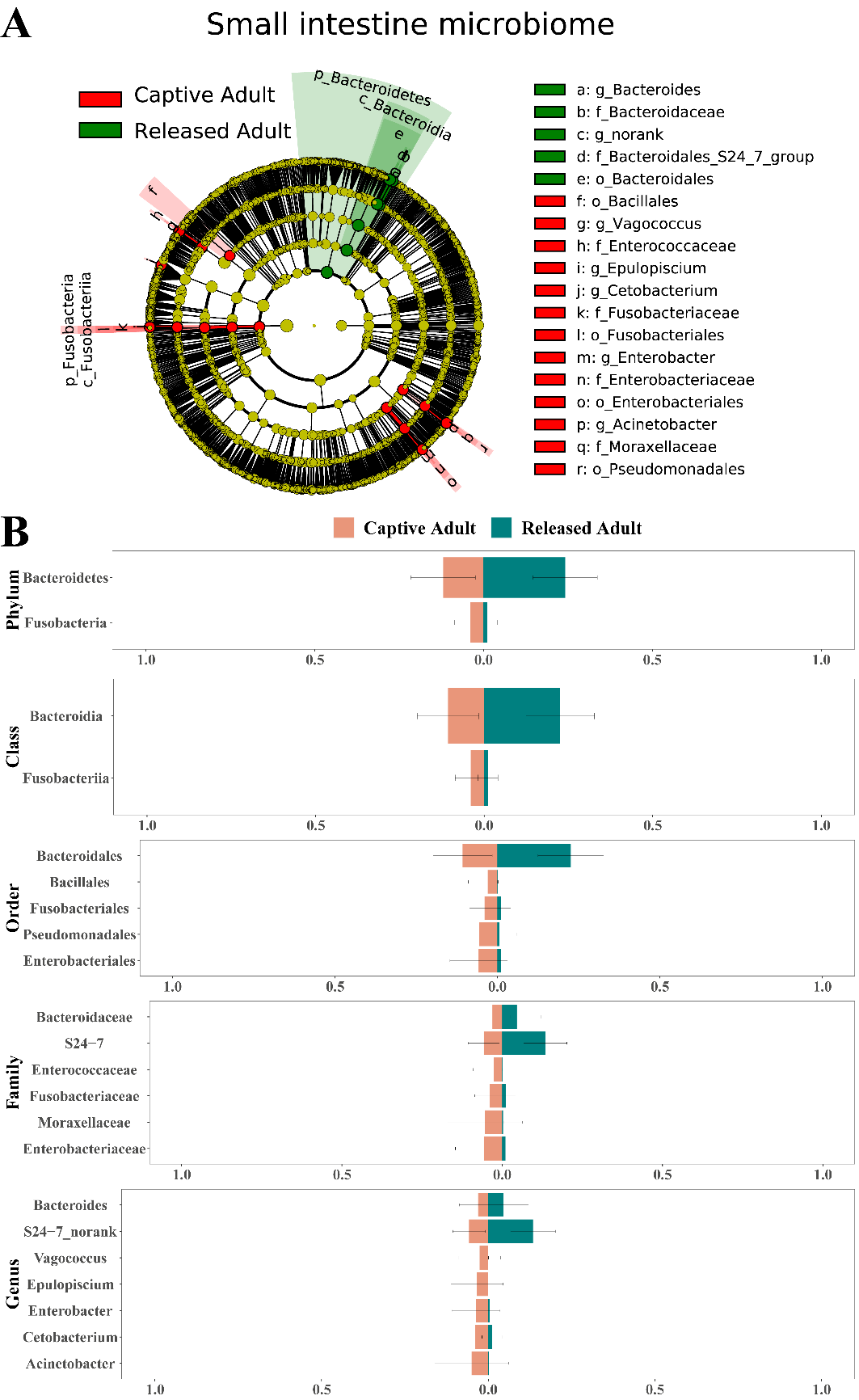


**Figure S7 |** Difference of the small intestine microbiota between captive bred and released adults. **(A)** A LEfSe analysis identifies different abundant small intestine bacterial taxa between captive bred and released adults. **(B)** Side-by-side comparison of the mean relative abundance of different abundant small intestine bacterial taxa between captive bred and released adults at phylum, class, order, family, and genus levels. Data are presented as means ± SE in bar graphs.


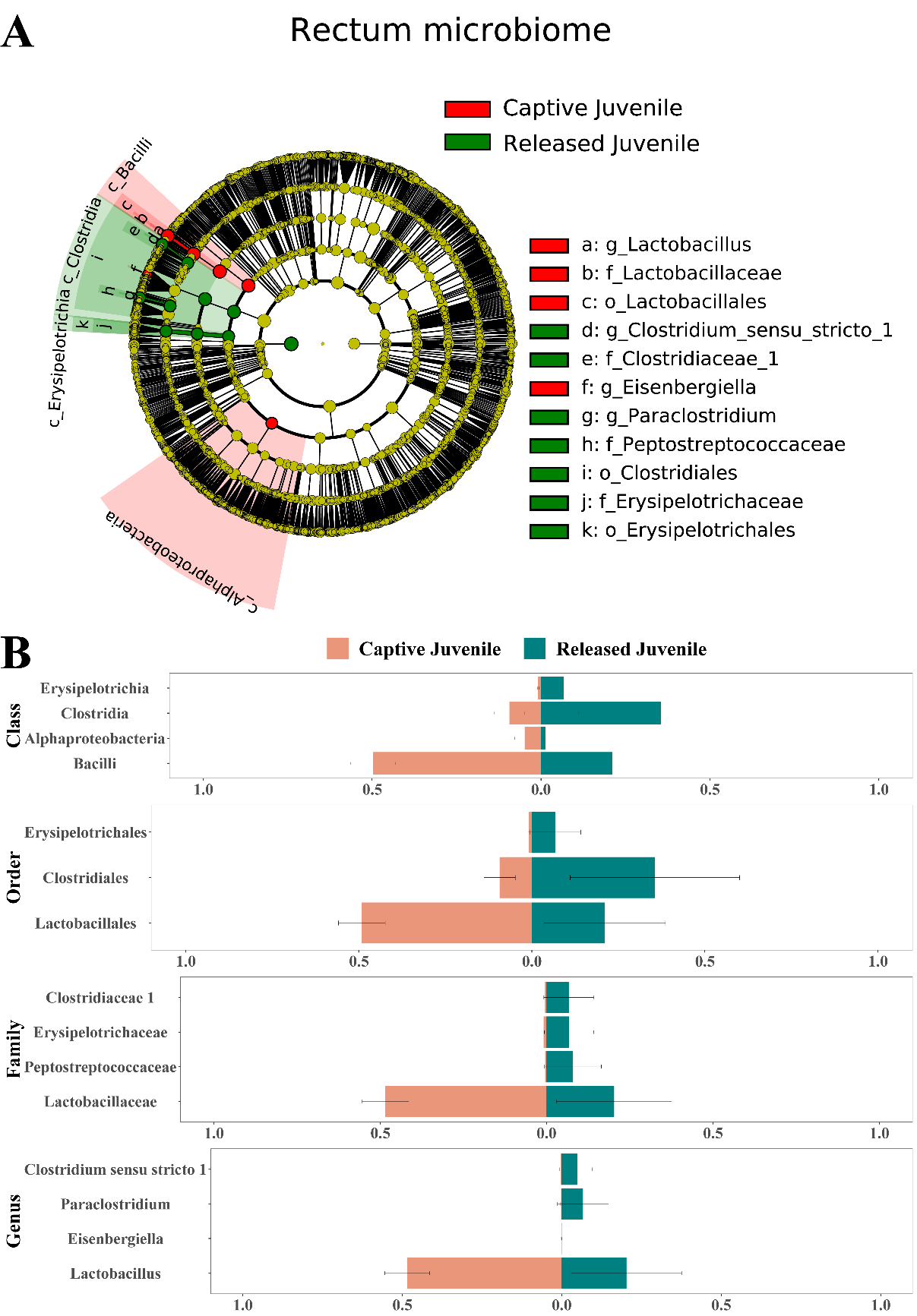


**Figure S8 |** Difference of the rectum microbiota between captive bred and released juveniles. **(A)** A LEfSe analysis identifies the different abundant rectum bacterial taxa between captive bred and released juveniles. **(B)** Side-by-side comparison of the mean relative abundance of different abundant rectum bacterial taxa between captive bred and released juveniles at phylum, class, order, family, and genus levels. Data are presented as means ± SE in bar graphs.


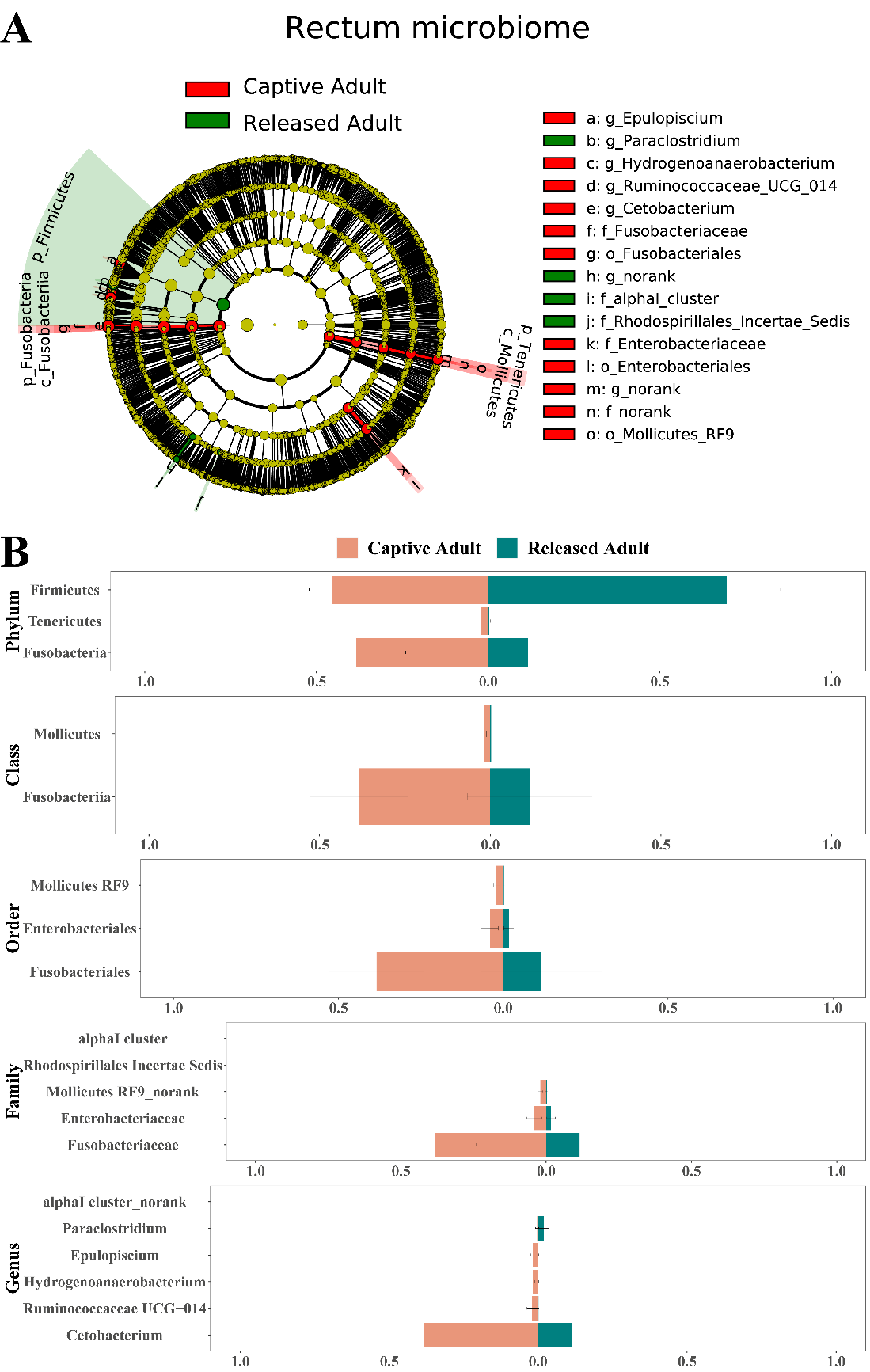


**Figure S9 |** Difference of the rectum microbiota between captive bred and released adults. **(A)** A LEfSe analysis identifies the different abundant rectum bacterial taxa between captive bred and released adults. **(B)** Side-by-side comparison of the mean relative abundance of different abundant rectum bacterial taxa between captive bred and released adults at phylum, class, order, family, and genus levels. Data are presented as means ± SE in bar graphs.


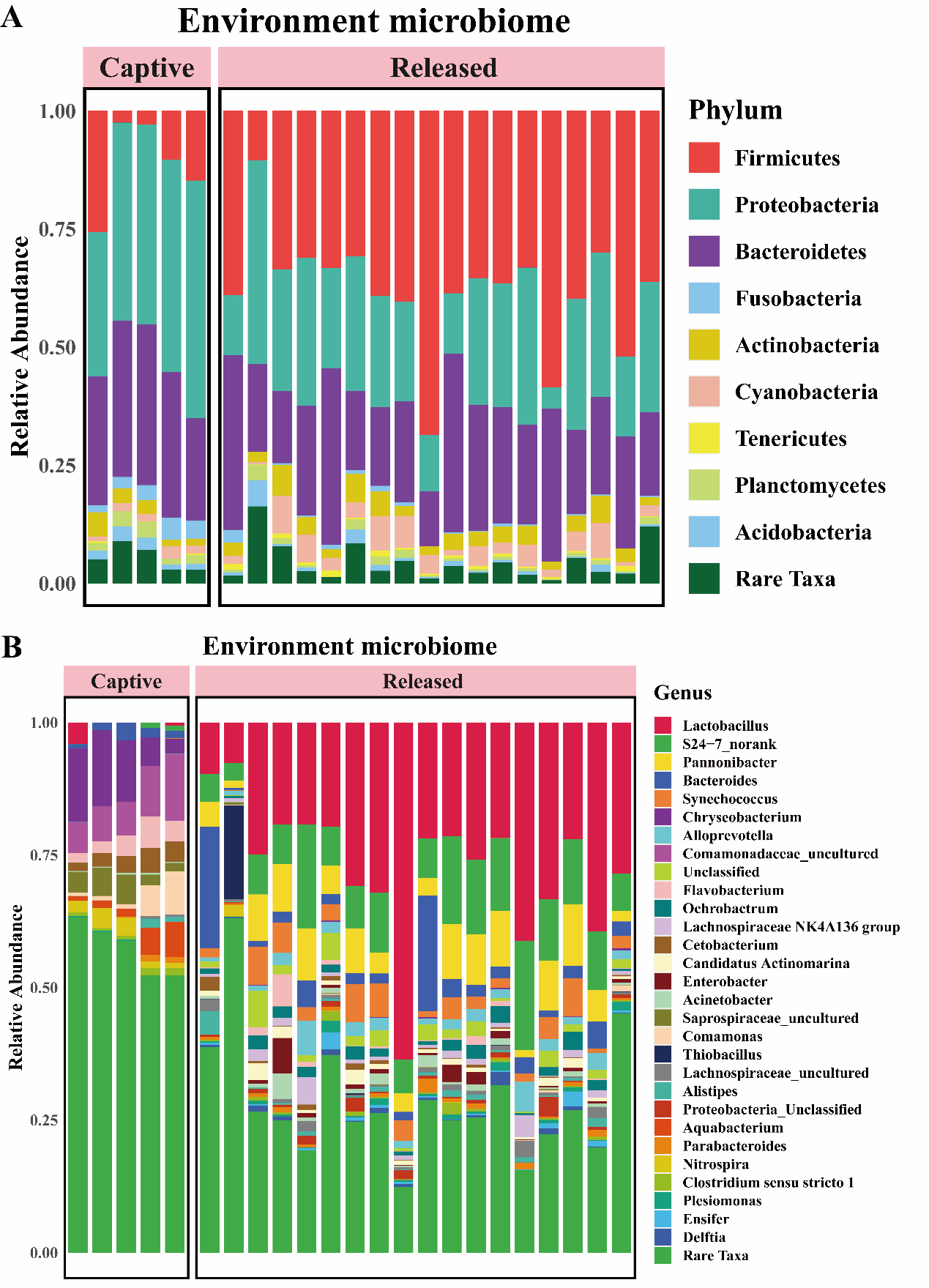


**Figure S10 |** Bar plots indicate the microbiota composition of water environment in hatchery and field at phylum **(A)** and genus level **(B)**.


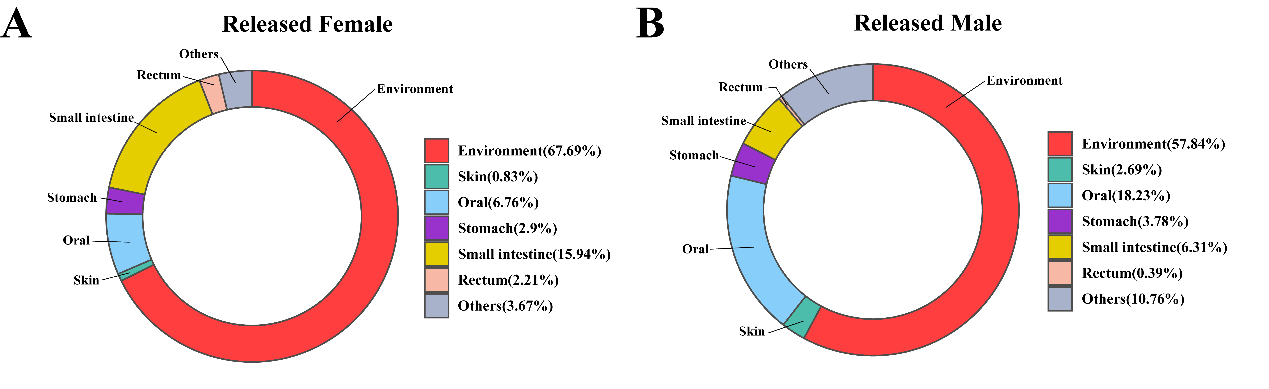


**Figure S 11 |** Source-tracking analyses reveal the reorganization of skin microbiota of released females **(A)** and males **(B)**. The environment and captive bred CGSs commensal microbiota are considered as the bacteria sources.
