# Supplementary material for: Reintroduction modifies the intraspecific variations of symbiotic microbes in captive bred Chinese giant salamander
Source: Front Microbiol. 2022 Dec 1;13:1062604. doi: 10.3389/fmicb.2022.1062604 (PMC9751345; doi:10.3389/fmicb.2022.1062604)
Supplement: Supplementary file 2 [file Data_Sheet_2.docx]

**Table S1 |** The comparisons of alpha diversity between captive bred female and male CGSs symbiotic microbiota. Observed OTU and Shannon diversity values are presented as means ± SE.

|  | Observed OTU | | | Shannon index | | |
| --- | --- | --- | --- | --- | --- | --- |
|  | Captive | | *P* | Captive | | *P* |
|  | Female | Male |  | Female | Male |  |
| Skin | 390.3±125.2 | 392.3±65.0 | 0.690 | 3.2±0.4 | 3.4±0.3 | 0.390 |
| Oral | 765.3±250.5 | 779.8±523.5 | 0.890 | 4.7±0.4 | 4.3±0.8 | 0.480 |
| Stomach | 550.0±212.1 | 404.8±22.7 | 0.340 | 3.5±0.6 | 3.3±0.4 | 0.590 |
| Small intestine | 470.0±106.2 | 525.4±271.8 | 0.960 | 3.2±1.2 | 3.6±0.9 | 0.400 |
| Rectum | 289.0±26.6 | 270.3±85.5 | 0.890 | 3.1±0.4 | 3±0.4 | 0.770 |

**Table S2** The results of statistical analysis for skin bacteria relative abundance at the family level between captive bred and released adults. Families showing significant difference between released and captive bred adults are in bold font.

|  | Skin microbiome | | | | | | | | | | | | | | | | | | Mann-Whitney U test |
| --- | --- | --- | --- | --- | --- | --- | --- | --- | --- | --- | --- | --- | --- | --- | --- | --- | --- | --- | --- |
|  | Captive Adult | | | | | | | | Released Adult | | | | | | | | | | P value |
| **Lactobacillaceae** | **0.00291** | **0.03572** | **0.05804** | **0.01291** | **0.00461** | **0.00332** | **0.00023** | **0.01597** | **0.40258** | **0.28848** | **0.23894** | **0.43060** | **0.25449** | **0.36637** | **0.46515** | **0.43317** | **0.48962** | **0.48478** | **<0.001** |
| **Moraxellaceae** | **0.44823** | **0.43264** | **0.22931** | **0.34693** | **0.38102** | **0.32914** | **0.42006** | **0.26605** | **0.00615** | **0.04388** | **0.05003** | **0.00215** | **0.00785** | **0.00362** | **0.01250** | **0.00227** | **0.01012** | **0.01265** | **<0.001** |
| **Enterobacteriaceae** | **0.13563** | **0.18596** | **0.16754** | **0.18502** | **0.06951** | **0.13219** | **0.10085** | **0.12019** | **0.01155** | **0.08779** | **0.01129** | **0.01340** | **0.01174** | **0.01363** | **0.00812** | **0.01812** | **0.01854** | **0.01680** | **<0.001** |
| **S24-7** | **0.00049** | **0.00389** | **0.01325** | **0.00393** | **0.00072** | **0.00347** | **0.00087** | **0.01257** | **0.12015** | **0.12589** | **0.06774** | **0.10822** | **0.10742** | **0.13620** | **0.08171** | **0.07269** | **0.06740** | **0.10761** | **<0.001** |
| **Flavobacteriaceae** | **0.05509** | **0.02485** | **0.01982** | **0.02401** | **0.19193** | **0.07741** | **0.07235** | **0.03478** | **0.01186** | **0.07858** | **0.01639** | **0.01552** | **0.01692** | **0.00929** | **0.02182** | **0.01937** | **0.01371** | **0.01748** | **0.003** |
| **Pseudomonadaceae** | **0.06449** | **0.09047** | **0.06181** | **0.05890** | **0.04591** | **0.07269** | **0.06744** | **0.08571** | **0.00604** | **0.00347** | **0.01114** | **0.00283** | **0.00351** | **0.00279** | **0.00536** | **0.01035** | **0.00702** | **0.00049** | **<0.001** |
| **Family XII** | **0.03398** | **0.03383** | **0.08469** | **0.06578** | **0.07695** | **0.08243** | **0.06211** | **0.04992** | **0.00166** | **0.00015** | **0.01473** | **0.00008** | **0.00748** | **0.00000** | **0.00004** | **0.00019** | **0.00060** | **0.00136** | **<0.001** |
| **Rhodobacteraceae** | **0.00140** | **0.00162** | **0.00555** | **0.00064** | **0.00042** | **0.00049** | **0.00008** | **0.00053** | **0.04761** | **0.03436** | **0.04720** | **0.04206** | **0.01363** | **0.08677** | **0.04742** | **0.06370** | **0.01643** | **0.03904** | **<0.001** |
| Ruminococcaceae | 0.01367 | 0.01986 | 0.02062 | 0.02322 | 0.00506 | 0.03281 | 0.02813 | 0.05513 | 0.02771 | 0.01737 | 0.00819 | 0.02492 | 0.01322 | 0.02972 | 0.02715 | 0.01189 | 0.03270 | 0.01676 | 0.633 |
| **Bacteroidaceae** | **0.00438** | **0.00933** | **0.01756** | **0.01635** | **0.00193** | **0.02605** | **0.00593** | **0.02201** | **0.03923** | **0.03379** | **0.01820** | **0.02907** | **0.03708** | **0.03115** | **0.04346** | **0.01665** | **0.01812** | **0.02300** | **0.003** |

**Table S3** The results of statistical analysis for skin bacteria relative abundance at the genus level between captive bred and released adults. Genera showing significant difference between released and captive bred adults are in bold font.

|  | Skin microbiome | | | | | | | | | | | | | | | | | | | Mann-Whitney U test | |
| --- | --- | --- | --- | --- | --- | --- | --- | --- | --- | --- | --- | --- | --- | --- | --- | --- | --- | --- | --- | --- | --- |
|  | Captive bred Adults | | | | | | | | Released Adults | | | | | | | | | | | ***P* value** |  |
| **Lactobacillus** | **0.00291** | **0.03572** | **0.05804** | **0.01291** | **0.00461** | **0.00332** | **0.00023** | **0.01597** | | **0.40258** | **0.28848** | **0.23894** | **0.43060** | **0.25449** | **0.36637** | **0.46515** | **0.43317** | **0.48962** | **0.48478** | **<0.001** |  |
| **Acinetobacter** | **0.42218** | **0.42029** | **0.22130** | **0.32699** | **0.36834** | **0.32114** | **0.40640** | **0.25536** | | **0.00608** | **0.04252** | **0.04837** | **0.00110** | **0.00623** | **0.00351** | **0.01137** | **0.00106** | **0.00872** | **0.01186** | **<0.001** |  |
| **S24-7 _norank** | **0.00049** | **0.00389** | **0.01325** | **0.00393** | **0.00072** | **0.00347** | **0.00087** | **0.01257** | | **0.12015** | **0.12589** | **0.06774** | **0.10822** | **0.10742** | **0.13620** | **0.08171** | **0.07269** | **0.06740** | **0.10761** | **<0.001** |  |
| **Enterobacter** | **0.08345** | **0.10440** | **0.09311** | **0.10278** | **0.06695** | **0.12762** | **0.09678** | **0.11513** | | **0.00374** | **0.07359** | **0.00544** | **0.00317** | **0.00506** | **0.00627** | **0.00374** | **0.00770** | **0.01110** | **0.01254** | **<0.001** |  |
| **Pseudomonas** | **0.06449** | **0.09047** | **0.06181** | **0.05890** | **0.04591** | **0.07269** | **0.06744** | **0.08571** | | **0.00604** | **0.00347** | **0.01114** | **0.00283** | **0.00351** | **0.00279** | **0.00536** | **0.01035** | **0.00702** | **0.00049** | **<0.001** |  |
| **Exiguobacterium** | **0.03398** | **0.03383** | **0.08465** | **0.06578** | **0.07695** | **0.08243** | **0.06211** | **0.04992** | | **0.00166** | **0.00015** | **0.01473** | **0.00008** | **0.00748** | **0.00000** | **0.00004** | **0.00019** | **0.00060** | **0.00128** | **<0.001** |  |
| **Bacteroides** | **0.00438** | **0.00933** | **0.01756** | **0.01635** | **0.00193** | **0.02605** | **0.00593** | **0.02201** | | **0.03923** | **0.03379** | **0.01820** | **0.02907** | **0.03708** | **0.03115** | **0.04346** | **0.01665** | **0.01812** | **0.02300** | **0.003** |  |
| **Pannonibacter** | **0.00008** | **0.00140** | **0.00279** | **0.00026** | **0.00019** | **0.00045** | **0.00008** | **0.00015** | | **0.03965** | **0.03138** | **0.02957** | **0.03546** | **0.00615** | **0.08348** | **0.04263** | **0.05301** | **0.00759** | **0.03293** | **<0.001** |  |
| **Aeromonas** | **0.05985** | **0.04153** | **0.03738** | **0.03077** | **0.01397** | **0.02228** | **0.02039** | **0.01533** | | **0.00060** | **0.00147** | **0.01469** | **0.00812** | **0.02994** | **0.00204** | **0.00245** | **0.00408** | **0.00140** | **0.00121** | **<0.001** |  |
| **Chryseobacterium** | **0.02541** | **0.00891** | **0.00215** | **0.00721** | **0.14496** | **0.03674** | **0.04029** | **0.01533** | | **0.00011** | **0.00057** | **0.00261** | **0.00057** | **0.00057** | **0.00143** | **0.00181** | **0.00057** | **0.00193** | **0.00079** | **<0.001** |  |

**Table S4** The results of statistical analysis for small intestine bacteria relative abundance at the family level between captive bred and released juveniles. Families showing significant difference between released and captive bred juveniles are in bold font.

|  | Small intestine microbiome | | | | | | | | | | | | | | | | | | | | Mann-Whitney U test | |
| --- | --- | --- | --- | --- | --- | --- | --- | --- | --- | --- | --- | --- | --- | --- | --- | --- | --- | --- | --- | --- | --- | --- |
|  | Captive Juvenile | | | | Released Juvenile | | | | | | | | | | | | | | | | *P* value | |
| **Lactobacillaceae** | **0.01329** | **0.37891** | **0.31332** | **0.21096** | **0.64394** | **0.32703** | **0.59560** | **0.50608** | **0.59746** | **0.70699** | **0.23478** | **0.46534** | **0.63363** | **0.64832** | **0.54210** | **0.29406** | **0.64843** | **0.63699** | **0.73490** | **0.30539** | **0.016** |  |
| S24-7 | 0.00174 | 0.04663 | 0.12604 | 0.01544 | 0.10591 | 0.09107 | 0.13019 | 0.12974 | 0.13061 | 0.06510 | 0.08605 | 0.09436 | 0.08443 | 0.08926 | 0.08681 | 0.03663 | 0.05864 | 0.04844 | 0.04659 | 0.09538 | 0.122 |  |
| Ruminococcaceae | 0.01001 | 0.17788 | 0.06374 | 0.11818 | 0.00861 | 0.03232 | 0.01424 | 0.01359 | 0.01862 | 0.01306 | 0.06502 | 0.04369 | 0.01782 | 0.01280 | 0.00868 | 0.49419 | 0.00853 | 0.00952 | 0.00838 | 0.42018 | 0.211 |  |
| **Enterobacteriaceae** | **0.33171** | **0.23025** | **0.02862** | **0.58983** | **0.00589** | **0.14749** | **0.00400** | **0.02560** | **0.01072** | **0.00861** | **0.01314** | **0.00548** | **0.01076** | **0.00555** | **0.04195** | **0.00744** | **0.00355** | **0.00699** | **0.00646** | **0.01850** | **0.002** |  |
| Bacteroidaceae | 0.13582 | 0.00393 | 0.04014 | 0.00551 | 0.02503 | 0.01941 | 0.03013 | 0.03440 | 0.02503 | 0.01416 | 0.03293 | 0.09073 | 0.01159 | 0.00902 | 0.01903 | 0.00585 | 0.00944 | 0.01386 | 0.01076 | 0.00691 | 1.000 |  |
| **SubsectionI_FamilyI** | **0.00053** | **0.01688** | **0.00155** | **0.00850** | **0.03232** | **0.01299** | **0.01473** | **0.02945** | **0.02130** | **0.03108** | **0.00110** | **0.02462** | **0.02843** | **0.04335** | **0.05569** | **0.01907** | **0.06593** | **0.06698** | **0.05426** | **0.00887** | **0.011** |  |
| **Fusobacteriaceae** | **0.40277** | **0.00276** | **0.03542** | **0.00351** | **0.00015** | **0.00057** | **0.00042** | **0.00151** | **0.00196** | **0.00000** | **0.03697** | **0.00091** | **0.00204** | **0.00053** | **0.00011** | **0.00385** | **0.00196** | **0.00113** | **0.00004** | **0.00132** | **0.007** |  |
| Prevotellaceae | 0.00049 | 0.00963 | 0.04361 | 0.00427 | 0.04663 | 0.03062 | 0.05045 | 0.03863 | 0.02613 | 0.01454 | 0.02364 | 0.02768 | 0.01238 | 0.00906 | 0.02560 | 0.00699 | 0.00706 | 0.01099 | 0.00510 | 0.01612 | 0.211 |  |
| Lachnospiraceae | 0.01775 | 0.02590 | 0.07533 | 0.00344 | 0.01155 | 0.01352 | 0.01786 | 0.01918 | 0.03576 | 0.01567 | 0.04871 | 0.02519 | 0.01295 | 0.01356 | 0.01442 | 0.00415 | 0.00517 | 0.00612 | 0.00834 | 0.01352 | 0.437 |  |
| Clostridiaceae 1 | 0.05316 | 0.00060 | 0.00804 | 0.00208 | 0.00140 | 0.16746 | 0.00132 | 0.00166 | 0.00468 | 0.00253 | 0.02568 | 0.00136 | 0.00223 | 0.00147 | 0.00215 | 0.00638 | 0.00415 | 0.01692 | 0.00121 | 0.00117 | 0.820 |  |

**Table S5** The results of statistical analysis for small intestine bacteria relative abundance at the genus level between captive bred and released juveniles. Genera showing significant difference between released and captive bred juveniles are in bold font.

|  | Small intestine microbiome | | | | | | | | | | | | | | | | | | | | Mann-Whitney U test | |
| --- | --- | --- | --- | --- | --- | --- | --- | --- | --- | --- | --- | --- | --- | --- | --- | --- | --- | --- | --- | --- | --- | --- |
|  | Captive Juvenile | | | | Released Juvenile | | | | | | | | | | | | | | | | *P* value |  |
| **Lactobacillus** | **0.01329** | **0.37891** | **0.31332** | **0.21096** | **0.64394** | **0.32703** | **0.59560** | **0.50608** | **0.59746** | **0.70699** | **0.23478** | **0.46534** | **0.63363** | **0.64832** | **0.54210** | **0.29406** | **0.64843** | **0.63699** | **0.73490** | **0.30539** | **0.016** |  |
| S24-7 _norank | 0.00174 | 0.04663 | 0.12604 | 0.01544 | 0.10591 | 0.09107 | 0.13019 | 0.12974 | 0.13061 | 0.06510 | 0.08605 | 0.09436 | 0.08443 | 0.08926 | 0.08681 | 0.03663 | 0.05864 | 0.04844 | 0.04659 | 0.09538 | 0.122 |  |
| **Plesiomonas** | **0.17580** | **0.03406** | **0.00143** | **0.51457** | **0.00015** | **0.12966** | **0.00015** | **0.00011** | **0.00030** | **0.00076** | **0.00876** | **0.00072** | **0.00234** | **0.00072** | **0.00057** | **0.00106** | **0.00098** | **0.00227** | **0.00344** | **0.00125** | **0.011** |  |
| **Anaerotruncus** | **0.00177** | **0.14801** | **0.00442** | **0.06797** | **0.00023** | **0.00034** | **0.00034** | **0.00023** | **0.00132** | **0.00102** | **0.00121** | **0.00238** | **0.00162** | **0.00000** | **0.00008** | **0.00000** | **0.00026** | **0.00030** | **0.00026** | **0.41244** | **0.007** |  |
| Bacteroides | 0.13582 | 0.00393 | 0.04014 | 0.00551 | 0.02503 | 0.01941 | 0.03013 | 0.03440 | 0.02503 | 0.01416 | 0.03293 | 0.09073 | 0.01159 | 0.00902 | 0.01903 | 0.00585 | 0.00944 | 0.01386 | 0.01076 | 0.00691 | 1.000 |  |
| **Synechococcus** | **0.00053** | **0.01688** | **0.00155** | **0.00850** | **0.03198** | **0.01280** | **0.01473** | **0.02885** | **0.02122** | **0.03089** | **0.00083** | **0.02405** | **0.02794** | **0.04248** | **0.05513** | **0.01907** | **0.06574** | **0.06634** | **0.05426** | **0.00887** | **0.011** |  |
| **Cetobacterium** | **0.40277** | **0.00276** | **0.03542** | **0.00351** | **0.00015** | **0.00057** | **0.00042** | **0.00151** | **0.00174** | **0.00000** | **0.03697** | **0.00038** | **0.00151** | **0.00053** | **0.00011** | **0.00374** | **0.00196** | **0.00113** | **0.00004** | **0.00132** | **0.007** |  |
| **Anaerofilum** | **0.00177** | **0.01186** | **0.00151** | **0.04614** | **0.00000** | **0.02205** | **0.00000** | **0.00000** | **0.00000** | **0.00000** | **0.00008** | **0.00038** | **0.00011** | **0.00234** | **0.00004** | **0.39934** | **0.00026** | **0.00000** | **0.00030** | **0.00000** | **0.029** |  |
| **Hafnia-Obesumbacterium** | **0.15266** | **0.19480** | **0.00381** | **0.07084** | **0.00000** | **0.00000** | **0.00019** | **0.00000** | **0.00000** | **0.00000** | **0.00000** | **0.00000** | **0.00004** | **0.00000** | **0.00000** | **0.00000** | **0.00000** | **0.00000** | **0.00000** | **0.00053** | **<0.001** |  |
| Alloprevotella | 0.00038 | 0.00914 | 0.03972 | 0.00419 | 0.04138 | 0.02451 | 0.03765 | 0.02756 | 0.02239 | 0.01084 | 0.01782 | 0.01639 | 0.00895 | 0.00653 | 0.02194 | 0.00615 | 0.00608 | 0.00816 | 0.00389 | 0.01359 | 0.385 |  |

**Table S6** The results of statistical analysis for small intestine bacteria relative abundance at the family level between captive bred and released adults. Families showing significant difference between released and captive bred adults are in bold font.

| Small intestine microbiome |  | sample | Lactobacillaceae | **S24-7** | Lachnospiraceae | Ruminococcaceae | **Bacteroidaceae** | **Enterobacteriaceae** | Peptostreptococcaceae | **Moraxellaceae** | **Fusobacteriaceae** | **Prevotellaceae** |
| --- | --- | --- | --- | --- | --- | --- | --- | --- | --- | --- | --- | --- |
|  | Captive Adult | MF_2 | 0.35795 | **0.11022** | 0.01676 | 0.02118 | **0.00978** | **0.01393** | 0.00072 | **0.00551** | **0.00004** | **0.02209** |
|  |  | MF_3 | 0.01087 | **0.00872** | 0.00476 | 0.02081 | **0.00427** | **0.32786** | 0.00064 | **0.12664** | **0.10671** | **0.00200** |
|  |  | MF1_2 | 0.55279 | **0.06880** | 0.01276 | 0.00800 | **0.01152** | **0.01941** | 0.00230 | **0.01057** | **0.00434** | **0.00959** |
|  |  | MF1_3 | 0.52794 | **0.15232** | 0.05214 | 0.06291 | **0.02945** | **0.00921** | 0.00143 | **0.00049** | **0.00563** | **0.03247** |
|  |  | MF2_2 | 0.39756 | **0.08820** | 0.02371 | 0.02870 | **0.01895** | **0.01182** | 0.00880 | **0.02235** | **0.01095** | **0.01322** |
|  |  | MF2_3 | 0.16871 | **0.04641** | 0.30905 | 0.02401 | **0.00668** | **0.00918** | 0.26216 | **0.00495** | **0.00744** | **0.00782** |
|  |  | MF3_2 | 0.47191 | **0.11060** | 0.04682 | 0.02753 | **0.03357** | **0.00770** | 0.00128 | **0.00306** | **0.02077** | **0.00532** |
|  |  | MF3_3 | 0.19446 | **0.03428** | 0.03119 | 0.16319 | **0.04338** | **0.05762** | 0.01171 | **0.01926** | **0.12147** | **0.00819** |
|  |  | MF4_2 | 0.96605 | **0.00374** | 0.00159 | 0.00268 | **0.00287** | **0.00079** | 0.00030 | **0.00076** | **0.00060** | **0.00204** |
|  |  | MF4_3 | 0.00257 | **0.00208** | 0.00868 | 0.02224 | **0.00091** | **0.06819** | 0.00683 | **0.44774** | **0.01329** | **0.00038** |
|  |  | MF5_2 | 0.25302 | **0.05796** | 0.03912 | 0.05479 | **0.23603** | **0.00755** | 0.01831 | **0.00793** | **0.00200** | **0.00963** |
|  |  | MF5_3 | 0.00993 | **0.00242** | 0.11426 | 0.03470 | **0.03092** | **0.09221** | 0.55585 | **0.00026** | **0.09100** | **0.00060** |
|  |  | MF6_2 | 0.31143 | **0.11275** | 0.02636 | 0.02670 | **0.01710** | **0.01220** | 0.00083 | **0.00544** | **0.01722** | **0.01137** |
|  |  | MF6_3 | 0.12744 | **0.03330** | 0.09628 | 0.15130 | **0.01650** | **0.05871** | 0.01854 | **0.01518** | **0.10289** | **0.00642** |
|  |  | MF7_2 | 0.25106 | **0.08756** | 0.02088 | 0.05532 | **0.02330** | **0.01510** | 0.00868 | **0.00827** | **0.02730** | **0.02137** |
|  |  | MF7_3 | 0.00049 | **0.00019** | 0.02998 | 0.02500 | **0.00091** | **0.20499** | 0.05275 | **0.18007** | **0.09991** | **0.00008** |
|  | Released Adult | M_2 | 0.68959 | **0.10659** | 0.01076 | 0.01363 | **0.01503** | **0.00461** | 0.00068 | **0.00823** | **0.00030** | **0.02454** |
|  |  | M_3 | 0.38688 | **0.21485** | 0.09130 | 0.06204 | **0.03176** | **0.00699** | 0.00057 | **0.00336** | **0.00000** | **0.03213** |
|  |  | M1_2 | 0.56260 | **0.07431** | 0.01548 | 0.02443 | **0.07004** | **0.00329** | 0.00329 | **0.00193** | **0.00110** | **0.01163** |
|  |  | M1_3 | 0.23410 | **0.10236** | 0.03515 | 0.01971 | **0.01454** | **0.00359** | 0.00091 | **0.00030** | **0.00060** | **0.02296** |
|  |  | M2_2 | 0.63623 | **0.05309** | 0.00729 | 0.00691 | **0.01174** | **0.00468** | 0.00313 | **0.00211** | **0.00076** | **0.00857** |
|  |  | M2_3 | 0.47731 | **0.19797** | 0.06589 | 0.03851 | **0.02998** | **0.00551** | 0.00166 | **0.00366** | **0.00038** | **0.05592** |
|  |  | M3_2 | 0.39326 | **0.09092** | 0.03285 | 0.01386 | **0.01563** | **0.00498** | 0.00472 | **0.00113** | **0.00049** | **0.02284** |
|  |  | M3_3 | 0.39405 | **0.11860** | 0.02787 | 0.02424 | **0.03999** | **0.02435** | 0.01850 | **0.00389** | **0.00276** | **0.02182** |
|  |  | M4_2 | 0.32412 | **0.15243** | 0.03655 | 0.04274 | **0.03693** | **0.01771** | 0.00336 | **0.00483** | **0.01069** | **0.02832** |
|  |  | M4_3 | 0.34655 | **0.18151** | 0.03957 | 0.04089 | **0.03614** | **0.01238** | 0.00321 | **0.00340** | **0.00385** | **0.04780** |
|  |  | F_2 | 0.48388 | **0.10399** | 0.02934 | 0.04471 | **0.07929** | **0.00472** | 0.00140 | **0.00272** | **0.00083** | **0.03077** |
|  |  | F_3 | 0.37981 | **0.11917** | 0.03640 | 0.02213 | **0.01669** | **0.00325** | 0.00019 | **0.00068** | **0.00000** | **0.02715** |
|  |  | F1_2 | 0.40364 | **0.12192** | 0.01771 | 0.01680 | **0.03882** | **0.02681** | 0.01393 | **0.00242** | **0.00381** | **0.02839** |
|  |  | F1_3 | 0.34591 | **0.20948** | 0.04289 | 0.03557 | **0.03123** | **0.00982** | 0.00457 | **0.00366** | **0.00438** | **0.03327** |
|  |  | F2_2 | 0.43483 | **0.20008** | 0.07967 | 0.06766 | **0.03840** | **0.00457** | 0.00147 | **0.00057** | **0.00094** | **0.05324** |
|  |  | F2_3 | 0.35723 | **0.31053** | 0.02673 | 0.03670 | **0.01541** | **0.00257** | 0.00419 | **0.00068** | **0.07457** | **0.03961** |
|  |  | F3_2 | 0.33862 | **0.15591** | 0.05868 | 0.06374 | **0.03587** | **0.02356** | 0.01246 | **0.00113** | **0.00442** | **0.02749** |
|  |  | F3_3 | 0.40828 | **0.13401** | 0.03255 | 0.02847 | **0.04044** | **0.02598** | 0.02198 | **0.00359** | **0.00102** | **0.03678** |
|  |  | F4_2 | 0.56747 | **0.10954** | 0.05090 | 0.03062 | **0.01575** | **0.00393** | 0.00159 | **0.00136** | **0.00102** | **0.02349** |
|  |  | F4_3 | 0.00332 | **0.00155** | 0.06158 | 0.20344 | **0.00415** | **0.00091** | 0.00627 | **0.00000** | **0.12574** | **0.00019** |
|  |  | F5_2 | 0.29818 | **0.14643** | 0.03157 | 0.03319 | **0.02133** | **0.00918** | 0.00128 | **0.00529** | **0.00872** | **0.03629** |
|  |  | F5_3 | 0.13759 | **0.05014** | 0.04591 | 0.10761 | **0.37045** | **0.01763** | 0.00963 | **0.00389** | **0.00631** | **0.00555** |
|  | Mann-Whitney U test | *P* value | 0.064 | **<0.001** | 0.356 | 0.672 | **0.042** | **0.007** | 0.404 | **0.002** | **0.003** | **<0.001** |

**Table S7** The results of statistical analysis for small intestine bacteria relative abundance at the genus level between captive bred and released adults. Genera showing significant difference between released and captive bred adults are in bold font.

| Small intestine microbiome |  | sample | Lactobacillus | **S24-7 _norank** | **Bacteroides** | **Cetobacterium** | **Acinetobacter** | Synechococcus | **Alloprevotella** | **Enterobacter** | **Mycoplasma** | Ruminococcaceae_uncultured |
| --- | --- | --- | --- | --- | --- | --- | --- | --- | --- | --- | --- | --- |
|  | Captive Adult | MF_2 | 0.35795 | **0.11022** | **0.00978** | **0.00000** | **0.00487** | 0.01624 | **0.01756** | **0.00491** | **0.16912** | 0.00200 |
|  |  | MF_3 | 0.01087 | **0.00872** | **0.00427** | **0.10671** | **0.10863** | 0.00072 | **0.00200** | **0.24037** | **0.04678** | 0.00834 |
|  |  | MF1_2 | 0.55279 | **0.06880** | **0.01152** | **0.00423** | **0.00838** | 0.03357 | **0.00948** | **0.01080** | **0.04746** | 0.00143 |
|  |  | MF1_3 | 0.52794 | **0.15232** | **0.02945** | **0.00563** | **0.00034** | 0.01480 | **0.03164** | **0.00079** | **0.01778** | 0.00936 |
|  |  | MF2_2 | 0.39756 | **0.08820** | **0.01895** | **0.01095** | **0.02130** | 0.05788 | **0.01050** | **0.00706** | **0.02481** | 0.00449 |
|  |  | MF2_3 | 0.16871 | **0.04641** | **0.00668** | **0.00744** | **0.00351** | 0.02568 | **0.00691** | **0.00155** | **0.00619** | 0.00321 |
|  |  | MF3_2 | 0.47191 | **0.11060** | **0.03357** | **0.02077** | **0.00287** | 0.00072 | **0.00514** | **0.00302** | **0.00268** | 0.00502 |
|  |  | MF3_3 | 0.19446 | **0.03428** | **0.04338** | **0.12136** | **0.01665** | 0.05441 | **0.00615** | **0.00978** | **0.00117** | 0.07295 |
|  |  | MF4_2 | 0.96605 | **0.00374** | **0.00287** | **0.00060** | **0.00072** | 0.00034 | **0.00121** | **0.00053** | **0.00011** | 0.00045 |
|  |  | MF4_3 | 0.00257 | **0.00208** | **0.00091** | **0.01329** | **0.42395** | 0.00042 | **0.00030** | **0.06483** | **0.00536** | 0.01061 |
|  |  | MF5_2 | 0.25302 | **0.05796** | **0.23603** | **0.00200** | **0.00506** | 0.05211 | **0.00834** | **0.00385** | **0.01329** | 0.01212 |
|  |  | MF5_3 | 0.00993 | **0.00242** | **0.03092** | **0.09100** | **0.00026** | 0.00291 | **0.00030** | **0.01548** | **0.00412** | 0.00940 |
|  |  | MF6_2 | 0.31143 | **0.11275** | **0.01710** | **0.01722** | **0.00483** | 0.01129 | **0.01038** | **0.00302** | **0.01926** | 0.00653 |
|  |  | MF6_3 | 0.12744 | **0.03330** | **0.01650** | **0.10289** | **0.01216** | 0.03715 | **0.00581** | **0.02462** | **0.02073** | 0.08715 |
|  |  | MF7_2 | 0.25106 | **0.08756** | **0.02330** | **0.02730** | **0.00691** | 0.01473 | **0.01363** | **0.00672** | **0.01023** | 0.03262 |
|  |  | MF7_3 | 0.00049 | **0.00019** | **0.00091** | **0.09991** | **0.17905** | 0.00000 | **0.00008** | **0.19049** | **0.00370** | 0.01454 |
|  | Released Adult | M_2 | 0.68959 | **0.10659** | **0.01503** | **0.00030** | **0.00785** | 0.02832 | **0.02239** | **0.00253** | **0.00083** | 0.00215 |
|  |  | M_3 | 0.38688 | **0.21485** | **0.03176** | **0.00000** | **0.00306** | 0.00910 | **0.02998** | **0.00487** | **0.00060** | 0.01269 |
|  |  | M1_2 | 0.56260 | **0.07431** | **0.07004** | **0.00106** | **0.00113** | 0.04788 | **0.00729** | **0.00094** | **0.00011** | 0.00336 |
|  |  | M1_3 | 0.23410 | **0.10236** | **0.01454** | **0.00060** | **0.00030** | 0.00121 | **0.02250** | **0.00042** | **0.00668** | 0.00261 |
|  |  | M2_2 | 0.63623 | **0.05309** | **0.01174** | **0.00076** | **0.00087** | 0.06302 | **0.00525** | **0.00219** | **0.00015** | 0.00068 |
|  |  | M2_3 | 0.47731 | **0.19797** | **0.02998** | **0.00038** | **0.00325** | 0.00993 | **0.05490** | **0.00110** | **0.00057** | 0.00668 |
|  |  | M3_2 | 0.39326 | **0.09092** | **0.01563** | **0.00049** | **0.00106** | 0.00283 | **0.02190** | **0.00038** | **0.00091** | 0.00291 |
|  |  | M3_3 | 0.39405 | **0.11860** | **0.03999** | **0.00272** | **0.00378** | 0.01782 | **0.01801** | **0.01076** | **0.00381** | 0.00449 |
|  |  | M4_2 | 0.32412 | **0.15243** | **0.03693** | **0.01019** | **0.00362** | 0.00472 | **0.02250** | **0.00525** | **0.00045** | 0.00940 |
|  |  | M4_3 | 0.34655 | **0.18151** | **0.03614** | **0.00355** | **0.00234** | 0.00744 | **0.04308** | **0.00536** | **0.02084** | 0.00823 |
|  |  | F_2 | 0.48388 | **0.10399** | **0.07929** | **0.00023** | **0.00238** | 0.01880 | **0.01918** | **0.00128** | **0.00442** | 0.00702 |
|  |  | F_3 | 0.37981 | **0.11917** | **0.01669** | **0.00000** | **0.00045** | 0.00634 | **0.02688** | **0.00008** | **0.08549** | 0.00419 |
|  |  | F1_2 | 0.40364 | **0.12192** | **0.03882** | **0.00381** | **0.00174** | 0.02137 | **0.02333** | **0.00993** | **0.00030** | 0.00378 |
|  |  | F1_3 | 0.34591 | **0.20948** | **0.03123** | **0.00438** | **0.00321** | 0.01408 | **0.02926** | **0.00344** | **0.00128** | 0.00714 |
|  |  | F2_2 | 0.43483 | **0.20008** | **0.03840** | **0.00094** | **0.00045** | 0.00279 | **0.05154** | **0.00053** | **0.00000** | 0.02111 |
|  |  | F2_3 | 0.35723 | **0.31053** | **0.01541** | **0.07457** | **0.00064** | 0.00091 | **0.03836** | **0.00083** | **0.00008** | 0.01246 |
|  |  | F3_2 | 0.33862 | **0.15591** | **0.03587** | **0.00442** | **0.00045** | 0.02401 | **0.02118** | **0.00706** | **0.00381** | 0.00480 |
|  |  | F3_3 | 0.40828 | **0.13401** | **0.04044** | **0.00102** | **0.00242** | 0.02632 | **0.03213** | **0.01159** | **0.00091** | 0.00506 |
|  |  | F4_2 | 0.56747 | **0.10954** | **0.01575** | **0.00053** | **0.00034** | 0.02930 | **0.01650** | **0.00008** | **0.00748** | 0.00242 |
|  |  | F4_3 | 0.00332 | **0.00155** | **0.00415** | **0.12574** | **0.00000** | 0.00011 | **0.00015** | **0.00011** | **0.00000** | 0.14582 |
|  |  | F5_2 | 0.29818 | **0.14643** | **0.02133** | **0.00872** | **0.00495** | 0.01450 | **0.03327** | **0.00585** | **0.08669** | 0.00446 |
|  |  | F5_3 | 0.13759 | **0.05014** | **0.37045** | **0.00631** | **0.00291** | 0.00362 | **0.00438** | **0.00427** | **0.00155** | 0.02851 |
|  | Mann-Whitney U test | *P* value | 0.064 | **<0.001** | **0.042** | **0.003** | **0.002** | 0.942 | **<0.001** | **0.012** | **0.004** | 0.421 |

**Table S8** The results of statistical analysis for rectum bacteria relative abundance at the family level between captive bred and released juveniles. Families showing significant difference between released and captive bred juveniles are in bold font.

|  | Rectum microbiome | | | | | | | | | | | |  |
| --- | --- | --- | --- | --- | --- | --- | --- | --- | --- | --- | --- | --- | --- |
|  | Captive Juvenile | | | | Released Juvenile | | | | | | | | Mann-Whitney U test |
| sample | TF1_3 | TF2_3 | TF3_3 | TF4_3 | T_4 | T1_4 | T2_4 | T3_4 | T4_4 | T5_4 | T6_4 | T7_4 | *P* value |
| **Lactobacillaceae** | **0.46968** | **0.48441** | **0.40628** | **0.57627** | **0.43479** | **0.17954** | **0.02266** | **0.21553** | **0.37003** | **0.36656** | **0.00563** | **0.03062** | **0.008** |
| S24-7 | 0.09081 | 0.12883 | 0.04263 | 0.08390 | 0.10395 | 0.05736 | 0.01061 | 0.09198 | 0.06030 | 0.22923 | 0.00706 | 0.03383 | 0.570 |
| Ruminococcaceae | 0.02488 | 0.05120 | 0.04350 | 0.02247 | 0.03074 | 0.04425 | 0.09425 | 0.02907 | 0.04089 | 0.04946 | 0.35561 | 0.11773 | 0.214 |
| Enterobacteriaceae | 0.01786 | 0.00276 | 0.01257 | 0.00793 | 0.01386 | 0.49622 | 0.06872 | 0.00498 | 0.00638 | 0.00921 | 0.00759 | 0.01907 | 0.570 |
| **Peptostreptococcaceae** | **0.00128** | **0.00083** | **0.00600** | **0.00774** | **0.02779** | **0.05486** | **0.20137** | **0.02277** | **0.21194** | **0.00276** | **0.00544** | **0.10723** | **0.048** |
| **Erysipelotrichaceae** | **0.00721** | **0.01159** | **0.00831** | **0.00725** | **0.00967** | **0.00789** | **0.13499** | **0.21496** | **0.01337** | **0.02466** | **0.07982** | **0.05739** | **0.028** |
| **Clostridiaceae 1** | **0.00215** | **0.00140** | **0.00793** | **0.00563** | **0.01329** | **0.01057** | **0.11350** | **0.07397** | **0.09749** | **0.00521** | **0.00291** | **0.21666** | **0.048** |
| Lachnospiraceae | 0.01918 | 0.09904 | 0.02250 | 0.03349 | 0.01945 | 0.00717 | 0.07646 | 0.03746 | 0.02401 | 0.08873 | 0.05630 | 0.03259 | 1.000 |
| Bacteroidaceae | 0.01424 | 0.02568 | 0.14080 | 0.02749 | 0.04727 | 0.01303 | 0.00143 | 0.01457 | 0.00861 | 0.03368 | 0.00997 | 0.00517 | 0.154 |
| Christensenellaceae | 0.00249 | 0.00091 | 0.00283 | 0.00313 | 0.00404 | 0.00019 | 0.08035 | 0.00846 | 0.03225 | 0.00291 | 0.04119 | 0.10750 | 0.073 |

**Table S9** The results of statistical analysis for rectum bacteria relative abundance at the genus level between captive bred and released juveniles. Genera showing significant difference between released and captive bred juveniles are in bold font.

|  | Rectum microbiome | | | | | | | | | | | | | Mann-Whitney  U test | | |
| --- | --- | --- | --- | --- | --- | --- | --- | --- | --- | --- | --- | --- | --- | --- | --- | --- |
|  | Captive Juvenile | | | | | Released Juvenile | | | | | | | | P value | |  |
| **Lactobacillus** | **0.46968** | **0.48441** | **0.40628** | **0.57627** |  | **0.43479** | **0.17954** | **0.02266** | **0.21553** | **0.37003** | **0.36656** | **0.00563** | **0.03062** | **0.008** |  |  |
| S24-7 _norank | 0.09081 | 0.12883 | 0.04263 | 0.08390 | | 0.10395 | 0.05736 | 0.01061 | 0.09198 | 0.06030 | 0.22923 | 0.00706 | 0.03383 | 0.570 |  |  |
| Plesiomonas | 0.00430 | 0.00083 | 0.00291 | 0.00370 | | 0.00751 | 0.48935 | 0.06683 | 0.00185 | 0.00374 | 0.00019 | 0.00045 | 0.01763 | 0.570 |  |  |
| **Paraclostridium** | **0.00011** | **0.00000** | **0.00083** | **0.00646** | | **0.00963** | **0.05184** | **0.18819** | **0.01159** | **0.19185** | **0.00166** | **0.00480** | **0.07193** | **0.016** |  |  |
| **Clostridium sensu stricto 1** | **0.00162** | **0.00140** | **0.00729** | **0.00393** | | **0.00559** | **0.00959** | **0.11279** | **0.07352** | **0.09198** | **0.00502** | **0.00287** | **0.08745** | **0.048** |  |  |
| Breznakia | 0.00060 | 0.00038 | 0.00121 | 0.00264 | | 0.00042 | 0.00015 | 0.12702 | 0.20416 | 0.00381 | 0.00385 | 0.00015 | 0.02337 | 0.461 |  |  |
| Ruminococcaceae_uncultured | 0.00680 | 0.00506 | 0.01084 | 0.00464 | | 0.00480 | 0.00038 | 0.06196 | 0.00793 | 0.00755 | 0.00823 | 0.20703 | 0.04187 | 0.368 |  |  |
| Bacteroides | 0.01424 | 0.02568 | 0.14080 | 0.02749 | | 0.04727 | 0.01303 | 0.00143 | 0.01457 | 0.00861 | 0.03368 | 0.00997 | 0.00517 | 0.154 |  |  |
| Christensenellaceae R-7 group | 0.00196 | 0.00057 | 0.00264 | 0.00219 | | 0.00276 | 0.00019 | 0.06932 | 0.00094 | 0.02454 | 0.00211 | 0.03187 | 0.10418 | 0.283 |  |  |
| Cetobacterium | 0.00570 | 0.00181 | 0.00744 | 0.00336 | | 0.03553 | 0.00004 | 0.01091 | 0.00038 | 0.00174 | 0.01556 | 0.00283 | 0.11158 | 1.000 |  |  |

**Table S 10** The results of statistical analysis for rectum bacteria relative abundance at the family level between captive bred and released adults. Families showing significant difference between released and captive bred adults are in bold font.

|  | Rectum microbiome | | | | | | | | | | | | | | | | | | | Mann-Whitney U test |
| --- | --- | --- | --- | --- | --- | --- | --- | --- | --- | --- | --- | --- | --- | --- | --- | --- | --- | --- | --- | --- |
|  | Captive Adult | | | | | | | | Released Adult | | | | | | | | | | | *P* value |
| **Fusobacteriaceae** | **0.36818** | **0.33367** | **0.40583** | **0.30837** | **0.13363** | **0.62702** | **0.50106** | **0.39258** | **0.54675** | **0.00253** | **0.00415** | **0.00004** | **0.37007** | **0.01337** | **0.17494** | **0.00249** | **0.00004** | **0.10750** | **0.04705** | **0.007** |
| Ruminococcaceae | 0.15530 | 0.23743 | 0.13552 | 0.23969 | 0.12713 | 0.10599 | 0.14545 | 0.18664 | 0.09330 | 0.02670 | 0.18932 | 0.28678 | 0.06155 | 0.02311 | 0.24849 | 0.46945 | 0.25989 | 0.37385 | 0.11951 | 0.840 |
| Erysipelotrichaceae | 0.10040 | 0.09296 | 0.15134 | 0.12015 | 0.14171 | 0.05052 | 0.09534 | 0.12936 | 0.14054 | 0.00778 | 0.27315 | 0.17739 | 0.04875 | 0.05592 | 0.24233 | 0.17777 | 0.28417 | 0.17554 | 0.24188 | 0.129 |
| Clostridiaceae 1 | 0.00982 | 0.07140 | 0.03289 | 0.01121 | 0.04395 | 0.02530 | 0.04452 | 0.04788 | 0.01008 | 0.11898 | 0.01597 | 0.07605 | 0.22183 | 0.00668 | 0.00132 | 0.01503 | 0.06325 | 0.00102 | 0.07193 | 1.000 |
| Lachnospiraceae | 0.04988 | 0.04278 | 0.08416 | 0.04742 | 0.03417 | 0.03330 | 0.05649 | 0.03746 | 0.02839 | 0.01559 | 0.04297 | 0.13789 | 0.02254 | 0.02390 | 0.05690 | 0.03119 | 0.03485 | 0.04969 | 0.03776 | 0.238 |
| Lactobacillaceae | 0.00748 | 0.00419 | 0.00570 | 0.00110 | 0.00072 | 0.00034 | 0.00053 | 0.00015 | 0.00174 | 0.23218 | 0.01280 | 0.00087 | 0.00215 | 0.47610 | 0.00140 | 0.00023 | 0.01216 | 0.00272 | 0.08730 | 0.091 |
| Christensenellaceae | 0.04829 | 0.00646 | 0.00838 | 0.03361 | 0.02688 | 0.02790 | 0.01752 | 0.01627 | 0.07042 | 0.00230 | 0.18396 | 0.04361 | 0.05037 | 0.01257 | 0.04810 | 0.02001 | 0.01948 | 0.14930 | 0.05618 | 0.109 |
| Peptostreptococcaceae | 0.01918 | 0.02379 | 0.04214 | 0.01760 | 0.02247 | 0.03836 | 0.05996 | 0.03104 | 0.03685 | 0.02073 | 0.06842 | 0.07688 | 0.13159 | 0.02224 | 0.06461 | 0.01212 | 0.05332 | 0.00487 | 0.06815 | 0.351 |
| Coriobacteriaceae | 0.06494 | 0.03553 | 0.02179 | 0.04308 | 0.02851 | 0.01575 | 0.01038 | 0.01744 | 0.00778 | 0.00615 | 0.10924 | 0.08122 | 0.04142 | 0.01038 | 0.03455 | 0.02462 | 0.04505 | 0.03070 | 0.02828 | 0.778 |
| Enterobacteriaceae | 0.01722 | 0.06094 | 0.04505 | 0.08462 | 0.02836 | 0.01948 | 0.01223 | 0.05600 | 0.00166 | 0.03968 | 0.00434 | 0.02300 | 0.02088 | 0.02451 | 0.00544 | 0.00193 | 0.03950 | 0.00143 | 0.02209 | 0.051 |

**Table S11** The results of statistical analysis for rectum bacteria relative abundance at the genus level between captive bred and released adults. Genera showing significant difference between released and captive bred adults are in bold font.

|  | Rectum microbiome | | | | | | | | | | | | | | | | | | | Mann-Whitney U test | |
| --- | --- | --- | --- | --- | --- | --- | --- | --- | --- | --- | --- | --- | --- | --- | --- | --- | --- | --- | --- | --- | --- |
|  | Captive Adult | | | | | | | | Released Adult | | | | | | | | | | | P value |  |
| **Cetobacterium** | **0.36818** | **0.33367** | **0.40583** | **0.30837** | **0.13363** | **0.62702** | **0.50106** | **0.39258** | **0.54675** | **0.00238** | **0.00415** | **0.00004** | **0.37007** | **0.01337** | **0.17494** | **0.00249** | **0.00004** | **0.10750** | **0.04678** | **0.007** |  |
| Ruminococcaceae_uncultured | 0.06823 | 0.12162 | 0.09523 | 0.10931 | 0.04561 | 0.04765 | 0.07926 | 0.11158 | 0.07269 | 0.01155 | 0.11547 | 0.22927 | 0.03557 | 0.00876 | 0.19272 | 0.31483 | 0.11996 | 0.26110 | 0.08269 | 0.492 |  |
| Faecalitalea | 0.04988 | 0.08764 | 0.12415 | 0.06525 | 0.03051 | 0.04607 | 0.07722 | 0.09545 | 0.04667 | 0.00060 | 0.02836 | 0.16538 | 0.02386 | 0.03957 | 0.04414 | 0.09772 | 0.21054 | 0.01125 | 0.09915 | 0.442 |  |
| Breznakia | 0.05022 | 0.00340 | 0.02647 | 0.05486 | 0.11105 | 0.00438 | 0.01782 | 0.03357 | 0.09353 | 0.00268 | 0.24317 | 0.01106 | 0.02481 | 0.00491 | 0.19793 | 0.06721 | 0.05037 | 0.16123 | 0.14122 | 0.238 |  |
| Lactobacillus | 0.00748 | 0.00419 | 0.00570 | 0.00110 | 0.00072 | 0.00034 | 0.00053 | 0.00015 | 0.00174 | 0.23218 | 0.01280 | 0.00087 | 0.00215 | 0.47610 | 0.00140 | 0.00023 | 0.01216 | 0.00272 | 0.08730 | 0.091 |  |
| Clostridium sensu stricto 1 | 0.00902 | 0.06989 | 0.03051 | 0.01091 | 0.04331 | 0.02247 | 0.04146 | 0.04599 | 0.00566 | 0.10346 | 0.01571 | 0.07578 | 0.13419 | 0.00665 | 0.00125 | 0.01499 | 0.06245 | 0.00094 | 0.07118 | 0.968 |  |
| Christensenellaceae R-7 group | 0.04418 | 0.00589 | 0.00612 | 0.02813 | 0.02613 | 0.02745 | 0.01080 | 0.01476 | 0.03678 | 0.00189 | 0.14061 | 0.04029 | 0.04418 | 0.01205 | 0.00559 | 0.01797 | 0.01729 | 0.14076 | 0.03749 | 0.351 |  |
| Coriobacteriaceae_uncultured | 0.06393 | 0.03372 | 0.02043 | 0.04301 | 0.02530 | 0.01525 | 0.00993 | 0.01643 | 0.00702 | 0.00423 | 0.10119 | 0.07861 | 0.03904 | 0.00566 | 0.03157 | 0.02262 | 0.04312 | 0.02602 | 0.02583 | 0.778 |  |
| Bacteroides | 0.02292 | 0.00933 | 0.00415 | 0.00272 | 0.00279 | 0.00502 | 0.01087 | 0.00736 | 0.01125 | 0.06740 | 0.00434 | 0.01110 | 0.00125 | 0.01348 | 0.00106 | 0.09889 | 0.07484 | 0.00653 | 0.00910 | 0.310 |  |
| [Eubacterium] coprostanoligenes group | 0.01110 | 0.02828 | 0.00427 | 0.01465 | 0.00672 | 0.00638 | 0.01858 | 0.00982 | 0.00808 | 0.00057 | 0.00325 | 0.01439 | 0.00366 | 0.00181 | 0.03070 | 0.06838 | 0.08677 | 0.02194 | 0.00751 | 0.904 |  |
